# (Not) Home alone: Antigen presenting cell – T Cell communication in barrier tissues

**DOI:** 10.3389/fimmu.2022.984356

**Published:** 2022-09-29

**Authors:** Teresa Neuwirth, Katja Knapp, Georg Stary

**Affiliations:** ^1^ Department of Dermatology, Medical University of Vienna, Vienna, Austria; ^2^ CeMM Research Center for Molecular Medicine of the Austrian Academy of Sciences, Vienna, Austria; ^3^ Ludwig Boltzmann Institute for Rare and Undiagnosed Diseases, Vienna, Austria

**Keywords:** skin, T cells, antigen-presenting cells, female reproductive tract, tissue-resident T cells, intestine, barrier tissue

## Abstract

Priming of T cells by antigen presenting cells (APCs) is essential for T cell fate decisions, enabling T cells to migrate to specific tissues to exert their effector functions. Previously, these interactions were mainly explored using blood-derived cells or animal models. With great advances in single cell RNA-sequencing techniques enabling analysis of tissue-derived cells, it has become clear that subsets of APCs are responsible for priming and modulating heterogeneous T cell effector responses in different tissues. This composition of APCs and T cells in tissues is essential for maintaining homeostasis and is known to be skewed in infection and inflammation, leading to pathological T cell responses. This review highlights the commonalities and differences of T cell priming and subsequent effector function in multiple barrier tissues such as the skin, intestine and female reproductive tract. Further, we provide an overview of how this process is altered during tissue-specific infections which are known to cause chronic inflammation and how this knowledge could be harnessed to modify T cell responses in barrier tissue.

## Introduction

**Figure 1:**
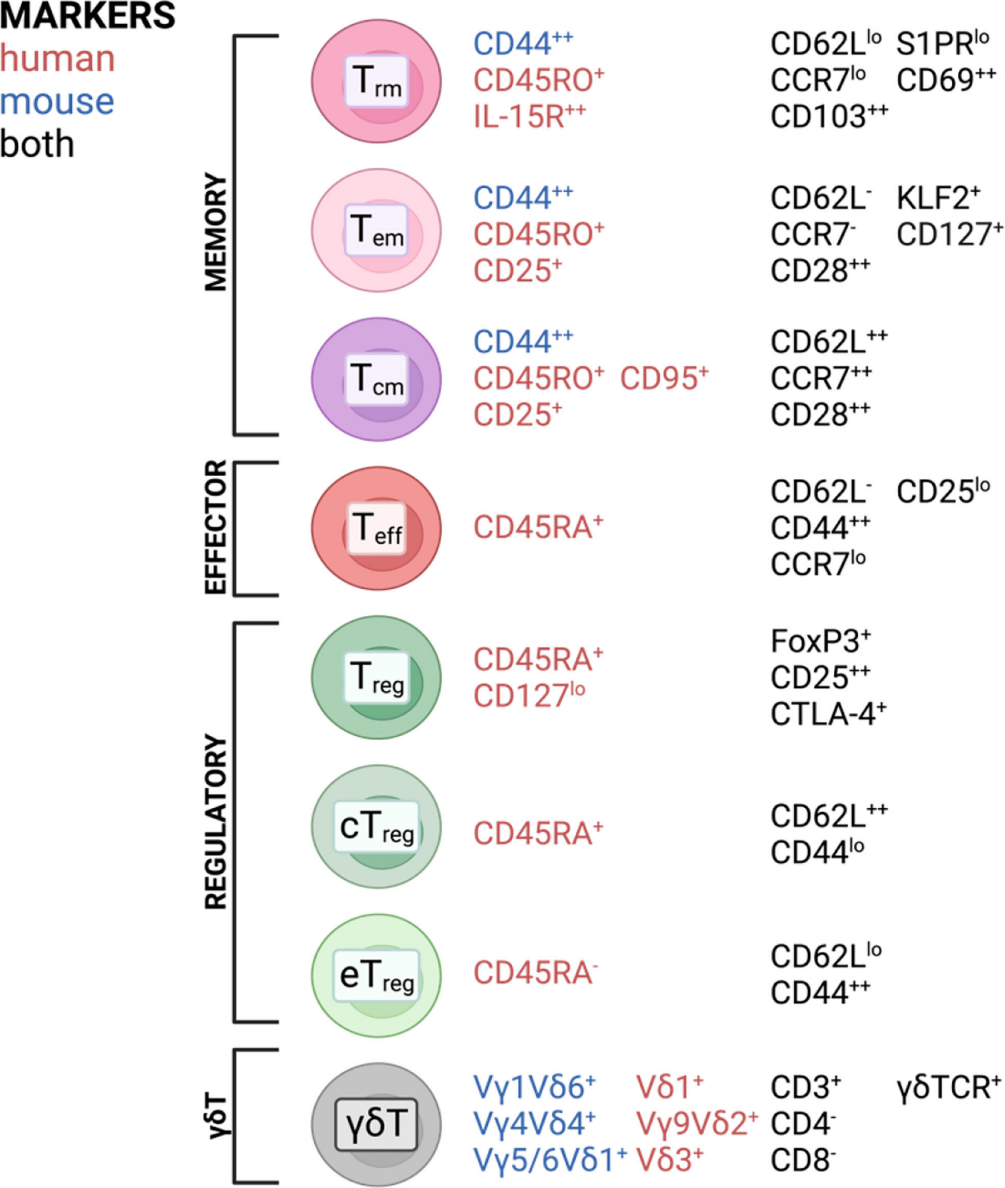
T cell subsets and commonly associated markers in mice and humans found in barrier tissues discussed in this review. Created with BioRender.com.

T cells are highly specialized executors of immune responses against pathogens and play important roles in maintaining tissue homeostasis. During infection or acute and chronic inflammatory responses, effector T cells (T_eff_) can infiltrate from the periphery and establish residency and subsequent memory, involving a switch in transcriptional program using different transcription factors and signaling hubs (1–6). This explains why the majority of the T cell population found in tissues are memory T cells (7), subdivided into central memory T (T_cm_), effector memory T (T_em_), and resident memory T (T_rm_) cells. T_em_ and T_cm_ were first identified in the peripheral blood (8). T_em_ were found to be the predominant subset in non-lymphoid tissue while their T_cm_ counterparts are mainly found in secondary lymphoid organs (9–17). Later, a long-lived memory population with little to no recirculatory capacity was identified and termed T_rm_ (12–18). Another prevalent T cell subset in tissues are regulatory T cells (T_regs_), particularly important for maintaining a tolerogenic tissue environment, preventing excessive immune responses to harmless antigens often found at barrier tissues [reviewed in (19, 20)]. T_regs_ usually refer to CD4^+^ T cells with the unique ability to suppress pro-inflammatory effector functions in other T cells as well as contribute to tissue homeostasis (21, 22). Tissue T_regs_ can also be subdivided by the central and effector memory cell classification based on the expression of CD44 and CD62L (23–25), with central T_regs_ being able to recirculate through secondary lymphoid tissues, while effector T_regs_ exhibit a more resident phenotype, representing the predominant T_reg_ population in nonlymphoid tissues (23). Non-conventional T cells can also be found in barrier tissues. An example of this are γδT cells, which are mainly found in epithelial tissues and are particularly abundant in the intestine (26). In homeostatic conditions, γδT cells have been described to exhibit a pre-activated memory phenotype (27), being able to exert direct cytotoxic functions (28, 29). As for other T cell subsets in tissues, roles in wound healing and tissue homeostasis have also been attributed to γδT cells (30, 31). A broad overview of T cell subsets found in tissues and surface markers most commonly associated with each subset is depicted in **Figure 1**. It should be noted that these markers are not absolute and their expression is often changed in different tissues. However, these figures aim to give a broad overview over the most common and widely distributed markers of each subset and highlight commonalities and differences between mice and humans.

Priming by antigen presenting cells (APCs) is crucial for T cells to exert their correct functions and home to tissues. For example, the presence and function of T_regs_ in tissue has been directly linked to the presence of dendritic cells (DCs) (32). Tissue-patrolling DCs are of an immature phenotype and internalize antigens by endocytosis or phagocytosis, which are loaded to major histocompatibility complex class II (MHC-II) for CD4 T cell presentation *via* endosomal pathways (33). However, DCs are also efficient in cross-presenting extracellular antigens *via* MHC-I to CD8 T cells, by which exact mechanism is still under debate (33, 34). Apart from antigen uptake, DCs need to receive additional stimuli in order to mature and upregulate CCR7, by which they interact with the ligands CCL19 and CCL21 guiding them to the lymph nodes to meet naïve T cells (35, 36). Under homeostatic conditions, DCs mainly collect non-hazardous antigens from food or commensal bacteria in the intestinal tract and skin or paternal antigens of fetal cells within the female genital tract during pregnancy (37–39). On the other hand, DCs are highly sensitive against pathogen-associated molecular patterns (PAMPs), which they detect *via* their toll-like receptors or C-type lectin receptors and they sense cytokines produced by other cell types during infection (33, 40). Mature DCs upregulate molecules necessary for co-stimulation of T cells like CD86 and CD80 (41).

**Figure 2:**
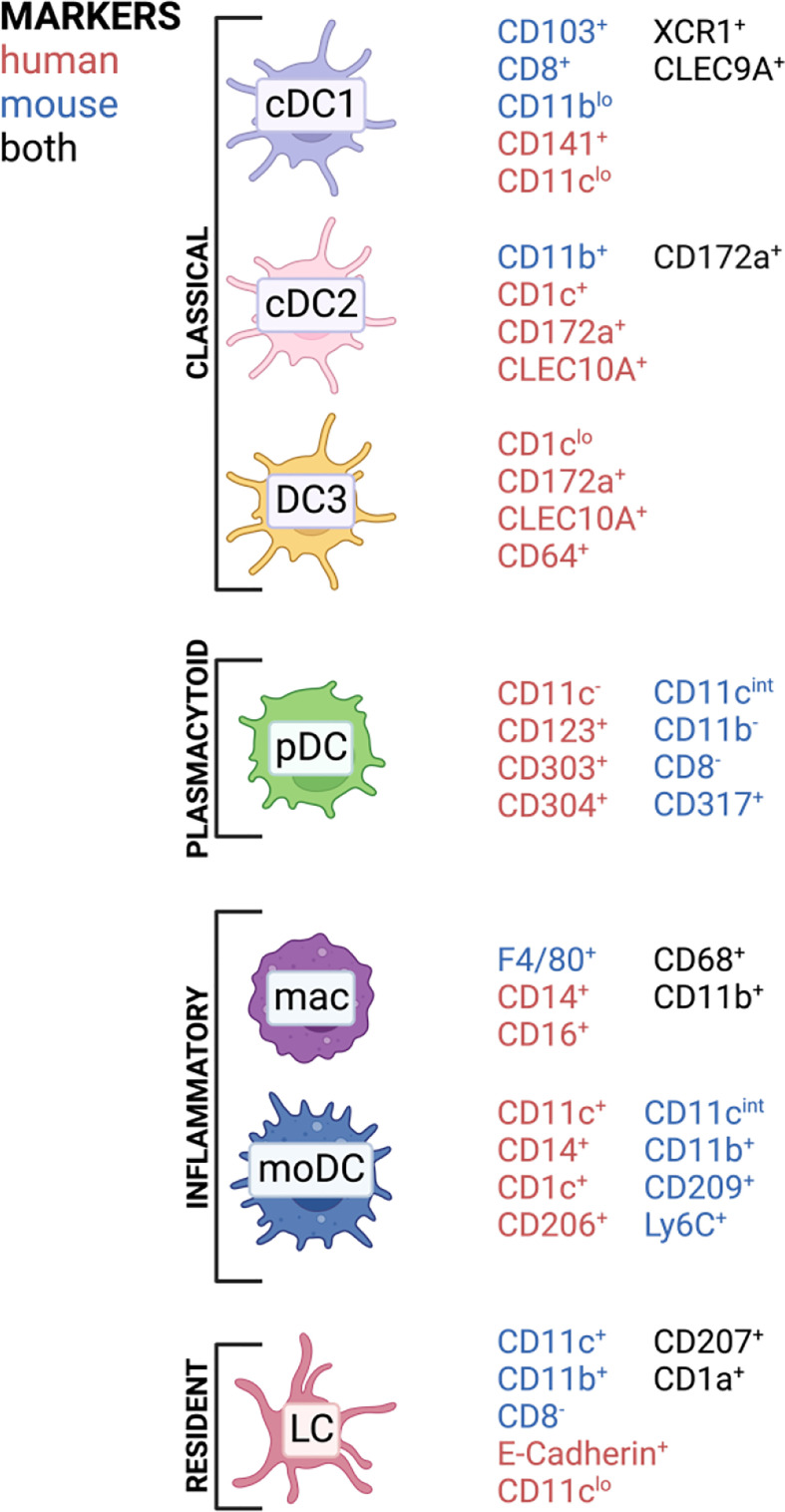
APC subsets and commonly associated markers in mice and humans found in barrier tissues discussed in this review. Created with BioRender.com.

Classically, DCs are divided into several subclasses: conventional DCs (cDCs), monocyte-derived DCs (mo-DCs) and plasmacytoid DCs (pDCs) (42). Langerhans cells were previously also classified as DC population; however, they developmentally originate from yolk sac progenitors, which identifies them as member of the tissue-resident macrophage family. In contrast to macrophages, they can efficiently present antigens and possess a migration potential to the lymph node (43). Therefore, they are often mentioned along with other DC subsets inducing T cell responses. Conventional DCs are subdivided into type 1 classical DC (cDC1), which are known to cross-present antigens *via* MHC-I to CD8 T cells but also polarize CD4 T cells towards T_h_1, while type 2 classical DCs (cDC2) mainly present antigens *via* MHC-II to CD4 T cells. The cDC1 subset in mice is CD11b^lo^ and shows CD8a and CD103 on their surface, while human cDC1 express and XCR1 and CD141 (33). cDC2 express CD172a and depending on murine or human origin they highly express CD11b or CD1c, respectively (33). Especially cDC2 comprises a very heterogenous immune cell population which can acquire quite contrary immune functions depending on the context. For examples, in human there exists a cDC2 subset which expresses at the same time monocyte-related genes like CD163 and CD14, which was termed DC3 (44, 45). LCs are a population patrolling the epidermis as well as the epithelial layer of the vagina and cervix and are characterized by expression of a specific lectin receptor, langerin (CD207) and CD1a (46). Monocytes express CD14 and can be differentiated *in vitro* to monocyte-derived DCs (mo-DCs) by addition of GM-CSF and IL-4 and are a widely used model for priming T cells *in vitro* (47). However, the existence of mo-DCs *in vivo* remains under debate, but several mouse (48, 49) and human (50) studies observed that monocytes can differentiate into DC-like cells, especially under inflammatory conditions (45, 51). With the evolving of single-cell sequencing technology, more and more DC subsets are discovered and it now appears that the discrimination between DC and monocyte subsets is not that black and white, with mo-DCs in comparison to DC3 being just one example (44, 45, 52, 53). APC subset composition varies widely throughout tissues and we are still far from understanding which subset contributes to immunity and tolerance under certain conditions (54–57). DCs are in general CD45^+^ cells, expressing HLA-DR and lacking other linage markers, such as CD3 or CD19 (52). In **Figure 2**, a simplified overview of the most important DC subsets in human can be found with the markers for those respective subsets in mice included.

In this review, we discuss the different subsets of T cells and APCs present in the skin, intestine and female reproductive tract (FRT) and how their interplay contributes to maintaining a homeostatic tissue environment as well as how this composition shifts during chronic inflammatory diseases and infection. While the term “immune homeostasis” is widely used, we refer to “homeostasis” as the balance between immune activation and suppression in tissues and organs in contribution to maintaining a healthy state of an organism under normal physiological conditions. This review aims to focus on the human system wherever possible; however, some insightful mechanistic studies in different animal models are included as these contribute greatly to our understanding of tissue immunity where human studies are not yet possible. To give a more comprehensive view of already described mechanistic studies not yet discovered in humans we also included animal studies when appropriate. Therefore, unless otherwise stated, findings summarized were done in humans and deviation to animal models is indicated.

## Skin

**Figure 3:**
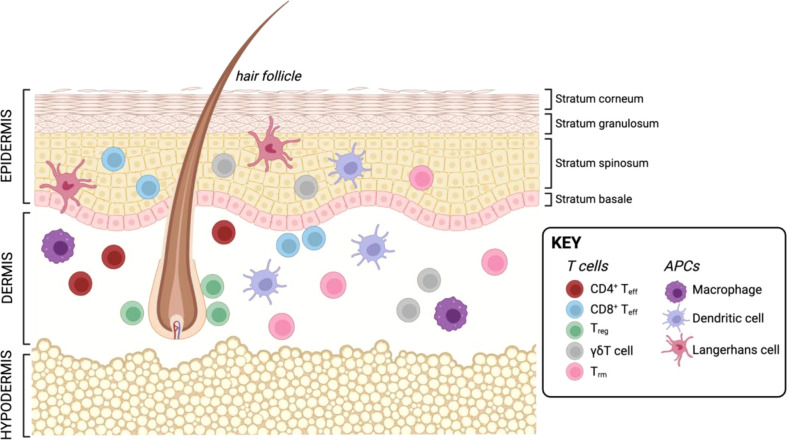
Resident T cells and APCs in the human skin. Created with BioRender.com.

The skin is one of the largest organs in the human body and essential for protection against external injury and pathogens. Next to its role in physical protection, the skin also houses a vast landscape of resident and recirculating immune cells which are poised locally to respond to tissue damage and infection. The skin is comprised of three layers: the outermost epidermis, an intermediate layer termed dermis, and the innermost layer called hypodermis (**Figure 3**). The epidermis is mainly comprised by structural cells such as keratinocytes, as well as melanocytes. The main immune cells found in this layer are CD8^+^ T cells and LCs, skin-resident macrophages which originate from the fetal liver and the yolk sac, and exhibit DC-like characteristics (58). Next to structural fibroblasts, the dermis contains the majority of immune cells, including DCs, macrophages, natural killer (NK) cells, innate lymphoid cells (ILCs), as well as CD4^+^ and CD8^+^ T cells. Further, this layer is also supplied with lymphatic and blood vessels which allow immune cell trafficking in and out of the tissue. The lowest layer, the hypodermis, is mainly comprised of adipocytes responsible for thermoregulation (59, 60). However, recently an immunological role has been attributed to adipose tissue as it has been shown to house multiple types of immune cells (61–66). Additionally, structures such as hair follicles and nerve endings are major players in regulating immune responses in the skin. Hair follicles represent unique structures in the skin, as many studies in mice have shown that they are the primary site for T_reg_ maintenance, which are in turn essential for establishing the stem cell niche at the hair follicle (67–69). In human skin, the hair follicle is also the major site of T_reg_ localization (70). Further, the hair follicle is also of importance for DC function in the skin of mice (68, 71). Besides the hair follicle, nerve endings in the skin have been shown to play an important role for CD8^+^ T cell mediated immunity (72) as well as create a special environment for specific macrophage subsets (73) as demonstrated in mouse models.

Upon encountering pathogens or injury to the epidermal layer, LCs are the first to initiate an immune response. These cells constitute approximately 2-4% of all cells in the epidermis and are specialized macrophages with DC characteristics, expressing the surface markers CD1a and Langerin/CD207 (46), whose dendrites can extend through the stratum corneum to sample antigen without disturbing the epithelial barrier (74, 75). LCs preferentially recognize mannosylated ligands on surfaces of pathogens *via* C-type lectins and pattern recognition receptors (PRRs) (76). Binding of these receptors leads to receptor-mediated endocytosis thereby activating the LC (77). Like their conventional DC counterpart, LCs have been found to be able to traffic to the skin-draining lymph nodes (LNs) and activate naïve T cells (78–80) as well as activate skin-resident T_regs_ (81). LCs have also been described to be highly efficient at inducing a neutralizing IgG response against *S. aureus* from B cells (82). While LCs have their primary role in immune surveillance of the skin, macrophages are mainly responsible for initiating inflammatory responses in response to infection or injury as well as to tissue regeneration (83–85).

Apart from the acute role of innate immune cells in clearing infection, APCs also play a major role in activating an adaptive immune response. As in other tissues, dermal APCs expressing CD1c (86, 87) can be divided into multiple subsets. In healthy human skin, the main subsets at steady-state are CD1a^++^CD207^++^ LCs, CD1a^+^CD1c^+^ DCs, CD141^++^CD14^-^ DCs, as well as two populations of macrophages that can be, in part, distinguished by their autofluorescence (AF) created by their high scatter properties: CD14^+^AF^-^ monocyte-derived macrophages (mo-Mac), and FXIIIA^+^CD14^+^AF^++^ macrophages (88). Upon antigen encounter in the skin, dermal DCs (DDCs) become migratory and act as APCs in the lymph node where they activate and polarize different adaptive immune cells, such as naïve T cells (88, 89). It was shown in mice that the constant travel of skin APCs to the LN during homeostasis is only dependent on the CCR7 ligand CCL21, whereas CCL19 presence is dispensable for the trafficking (90, 91). However, CCL19 deficient mice exhibit lower T cell numbers due to decreased cell survival (91). However, DCs in the skin have also been shown to locally activate memory T cells within the skin, bypassing the need for tissue egress (81) and thereby enabling a rapid adaptive immune response locally.

Specifically, T cells play a major role in the cutaneous immune system, with a large tissue-resident population being found throughout the dermis and epidermis. In healthy skin, this population can comprise up to 2x10^10^ cells, which is nearly two times as many as found in circulation (17). Differences in T cell composition between murine and human skin have made studies using mouse models difficult. In mice, the majority of resident T cells are γδT cells with a limited T cell receptor (TCR) repertoire (92), while in human skin most resident T cells are αβT cells with a much greater TCR diversity (17). Overall, T cells in the epidermis are less proliferative but have increased capacity to produce cytokines such as IFN-γ and TNF-α (93). While αβT cells rely on antigen presentation *via* MHC molecules, γδT cells have a restricted TCR repertoire, with their receptors recognizing unconventional antigens such as phosphoantigens, stress molecules, as well as non-peptide metabolites (94–96). Human γδT cells express the Vδ1, Vδ2, and Vδ3 chains, with each subtype having a preferential distribution across the body (97). A murine-specific γδT cell subset, called dendritic epidermal T cells (DETCs), have also been shown to significantly contribute to immune homeostasis in mouse skin (98), but don’t have a human counterpart. How different T cells subsets contribute to maintaining homeostasis and how this paradigm is shifted during the inflammatory response and infection will be discussed below.

### DC-T cell composition in homeostasis

#### Memory T cells

While the T cell subsets above mainly describe different effector states of activated T cells, a central part of T cell function is the capacity to develop long-lived immunological memory. T_eff_ cells primed in the lymph nodes by an APC are maintained in the skin as memory T cells, whose survival is supported by keratinocytes producing growth factors as well as other tissue resident (immune) cells (99, 100). These resident memory cells are crucial for maintaining tissue homeostasis as they contribute to immune surveillance and supply a rapid, specific response when re-encountering pathogens. As with all other immune cell subsets, memory T cells in the skin can be divided into two major groups: resident and recirculating. Using a human skin xenograft model with nude NSG mice, four distinct memory populations in the skin have been identified using the resident vs. recirculating paradigm. In human and mouse skin, the primarily resident subsets are T_em_ and T_rm_. Recirculating subsets can further be subdivided into migratory memory (T_mm_) and T_cm_ (8, 93, 101). Cutaneous lymphocyte antigen (CLA) is a marker that specifically distinguishes memory T cells originating from the cutaneous immune system as well as skin-infiltrating T cells. CLA binds to chemokine receptors, E-selectin which together with Very late antigen 1 (VLA-1)/Vascular cell adhesion protein 1 (VCAM-1) and Lymphocyte function-associated antigen 1 (LFA-1)/Intercellular adhesion molecule 1 (ICAM-1) enables skin tropism of these cells (102–105).

T_em_ are thought to be the first responders, expressing high levels of CD44 but lacking migratory and homing receptors such as L-selectin and CCR7 (8, 106, 107), making them incapable of recirculating. As their name suggests, they provide immediate effector function, which is underscored by their production of IFN-γ as well as other pro-inflammatory cytokines (93). While T_em_ are crucial for immediate adaptive responses, this population undergoes significant contraction after an infection is resolved and their niche has been found to be replaced by T_cm_ which enter from the circulation over the course of an acute inflammatory response (13). T_cm_ express high levels of homing receptors that are lacking on T_em_ (CCR7, LCA, CCR4) (17, 108, 109). Contrary to T_em_, their reactivation primarily takes place in the local LNs. There, they undergo extensive proliferation and adopt a T_em_-like phenotype (8, 110). The other circulating subset, T_mm_, was described by Rei et al. (93) and shows a population of cells high in skin-homing receptors such as CLA and CCR7 but are defined by the absence of L-selectin. This lack of L-selectin has raised suspicion that these cells are able to remain in the skin after infection, where they contribute to immune homeostasis as these cells are not high producers of pro-inflammatory cytokines (93). Another, more recently discovered, family member are T_rm_ which express high levels of the integrin CD103 as well as the glycoprotein CD69. While their overall phenotype is similar to that of T_em_, they have been shown to be maintained long-term even after an infection, as well as being significantly more potent in their effector response while also being limited in their proliferative capacity (13, 111). An essential tool in understanding the migratory behavior of T_rm_ is two-photon intravital microscopy. Multiple studies in mice have revealed that, in the skin, these cells are relatively stationary and confined in and close to the epidermis where they surveil their environment and are responsible for regulating secondary recall responses after primary challenge (112–114). Together, these memory subsets contribute to long-lasting immune memory and surveillance in the skin.

#### Effector T cells

While T cells in the skin at steady-state are mostly memory T cells, effector T cells (T_eff_) can also be found. These are activated by APCs in the skin-draining lymph nodes and traffic to the skin, where they further encounter cutaneous APCs presenting their cognate antigen, which leads to T cell activation and production of effector cytokines (115, 116). Most studies on T_eff_ cells have described essential roles for CD8^+^ T_eff_ cells in maintaining tissue homeostasis in the skin. CD8^+^ T cells can be found in both the dermis as well as the epidermis. CD8^+^ T cells have been shown to have increased migratory capacity within different skin compartments, albeit with slower kinetics than migration in the lymph node (117). In a sophisticated *ex vivo* imaging system of whole skin to observe T cell migration, Dijkgraaf et al., could demonstrate that human CD8^+^ skin-resident T_rm_ in the epidermis migrate along the stratum basale, close to the basement membrane and preferentially localize below aggregations of stationary LCs. In contrast, CD8^+^ T cells in the papillary dermis were observed to accumulate in collagen I rich regions as well as collagen I-poor dermal vessels. These observed migration dynamics highlight an important function of CD8^+^ skin-resident T cells in tissue patrol, possibly enabling immediate cytotoxic response to antigen presentation by co-localized LCs at the epidermal-dermal junction (118). While CD8^+^ T cell co-localization with LCs at the epidermis-dermis interface may hint at increased priming capacity by local epidermis-patrolling APCs, observed changes in morphology of CD8^+^ T_rm_ to a more dendrite-like shape (7, 117, 119, 120) could also suggest that these memory cells can act, at least in part, independently of APCs when confronted with their respective antigen, which has been described to be the case in mice (121–123). However, it is known that specialized CD141^+^CD103^+^ DCs are especially effective at cross-presentation for CD8^+^ T cells in the skin (124, 125).

#### Regulatory T cells

Similar to other immune cell populations, T_regs_ can reside in non-lymphoid tissue (NLT) such as the skin. Specific residency transcriptional programs in these organs have been described, mediating T_reg_ adaptability to different tissues in mice (126). In human skin, T_regs_ represent between 5% and 20% of all resident T cells under homeostatic conditions (127, 128), where they are known to interact with LCs and fibroblasts (81, 127). Most circulating T_regs_ found in peripheral blood express skin-homing markers which indicates that these cells are constitutively recruited to the skin over other organs (129). Similar to their effector memory counterparts, T_regs_ from the skin are also able to elicit a memory response and have been shown to persist in the skin and induce tolerance to autoantigens in a mouse model (130). In human skin, the function of skin-resident T_regs_ remains elusive, with few studies investigating their function under homeostatic conditions. Other than the canonical transcription factor FoxP3, skin T_regs_ express CLA, as well as the chemokine receptors CCR6, high levels of CCR4, a skin homing marker, high levels of L-selectin and HLA-DR. Similar to their blood counterparts, they express GITR and high levels of intracellular CTLA-4. Contrary to other skin-resident T_eff_ cells, skin T_regs_ tend to express much lower CD103 (127). Seneschal et al. demonstrated that the function of skin-resident T_regs_ is highly dependent upon the context under which they are activated by local LCs. Under steady-state, LCs appear to preferentially activate and expand CD4^+^CD25^+^FoxP3^+^CD127^-^ T_regs_, which were functionally competent in suppressing autologous skin resident T_em_ cells. Further, it was suggested that this effect is MHC-restricted, showing that under steady-state conditions, LCs act in concert with T_regs_ to induce and maintain tissue homeostasis (81). While reports of antigen-specific responses by T_regs_ do exist, it is well-established that skin T_regs_ have a high proliferative capacity in response to non-antigen dependent stimuli, such as contact with dermal fibroblasts in combination with IL-15 (127). Other than their immediate immunological function, cutaneous T_regs_ are known to be involved in wound healing (131, 132), where their primary role lies in inhibiting IFN-γ production by other T cells and inflammatory macrophages (132), as well as and modulating hair follicle stem cells (133).

#### γδT cells

In human skin, 1-10% of all resident T cells are estimated to be γδT cells (134), with the majority expressing the Vδ1 TCR (135, 136). One known ligand for this TCR is CD1d which is able to present lipid antigens on DCs (137). CCR6 on γδT cells is thought to be an important receptor mediating recruitment of activated γδT cells *via* CCL20 expression by keratinocytes, DCs as well as endothelial cells (138). CCL20 secretion by keratinocytes is especially upregulated during acute injury, suggesting an important role for γδT cells in response to injury (139). Cytokines important in αβT cell maintenance in the skin have also been found to play key roles for γδT cell maintenance and development in this organ. IL-7R signaling, for example, has been shown to induce rearrangement and transcription of the TCR γ-chain, and IL-15 is also involved in the expansion of γδ epidermal T cell precursors as well as their survival, while IL-4 signaling has been shown to promote growth of the epidermal γδT cell compartment (140–142). The skin residency marker CD103 has also been implicated to play a role in establishing γδT cells in the murine epidermis, with CD103-deficient mice showing significant reduction in γδT cell numbers in the skin as well as abrogated morphology in the γδT cells present (143). Further, murine CD103^-^ DETCs share a competitive niche in the epidermis with CD103^+^ T_rm_, indicating that CD103 is an important determinant in establishing tissue residency in the murine epidermis (113). If CD103 expression by γδT cells is also vital in human skin remains to be uncovered. Co-stimulation for γδT cells is less understood than for their αβ counterparts. However, in mice CD27 has been shown to contribute to the function of Vγ2Vδ2 T cell activation and promote IFN-γ production by these cells (144). Further CD2 and ICAM-1 have been suggested as costimulatory molecules for Vδ1 T cells (145–147). Specific functions of γδT cells in human skin are known to include regulation of keratinocyte proliferation and homeostasis through the production of insulin-growth factor 1 (IGF-1) and other keratinocyte growth factors (98, 148). Further, γδT cells are also able to contribute to skin homeostasis by recognizing damaged cells and exhibit cytotoxic activity *via* the NKG2D receptor (149), as well as perforin secretion and Fas-mediated cell lysis (150).

### DC-T cell composition in infection and inflammation

#### Chronic inflammatory diseases

A skewed composition in terms of T cell numbers and function of skin-resident T cells has been described in a plethora of chronic inflammatory skin diseases. Accordingly, the populations of APCs in inflamed skin also shift, with the dominant subsets being FcER1^+^CD1a^lo^ (inflammatory dendritic epidermal) DCs, CD1c^+^CD14^+^/^-^ DC (inflammatory), TNF-α^+^INOS^+^CD14^-^CD11c^+^CD1c^-^ (TNF-α and iNOS producing) DCs, and CD123^+^ pDCs depending on the nature of the disease (88). Further, in a mouse model of skin inflammation, Chow et al. demonstrated that specifically usually resting T_regs_ become highly motile during both adaptive and innate inflammation, highlighting the importance of these cells to control local inflammation (151).

One prominent example of such a disease is psoriasis, which affects 2-3% of the population (152). Skin lesions in psoriasis are thought to be caused by dysregulated cross-talk between APCs and T cells, which leads to an increased production of pro-inflammatory cytokines such as IL-17, IL-12, IFN-γ, TNF-α, and IL-23 (153, 154). This creates a positive feedback loop by recruiting more lymphocytes, neutrophils and myeloid cells to the lesion ultimately causing chronic cutaneous inflammation and epidermal hyperplasia (155). Blocking of TNF-α significantly reduced expression of the DC migration marker CCR7 and its ligand CCL19, thereby supporting clinical remission of patients (156). Dermal CD3^+^ T cells in these skin lesions are often increased by up to 15%. The composition of αβ and γδT cells in psoriasis also shifts, with some studies observing more than 40% of CD3^+^ T cells also expressing γδ TCRs and secreting the pathogenic cytokines IL-17 and IL-23 (157). Other studies have observed CLA^+^ Vγ2Vδ2 T cells homing to the skin to be increased in patients with psoriasis (158). Further, LCs have been described to preferentially utilize the MAPK-p38α signaling pathway, which has been linked to psoriasis susceptibility in humans (159). This has been shown to specifically promote production of IL-17 in CD4^+^ T cells by promoting the expression of IL-23 and IL-6, both of which are essential for T_h_17 differentiation and known to drive psoriasis pathogenesis (160). Additionally, LCs are able to induce a peripheral T cell response by priming immature CD4^+^ T cells in the lymph node to produce IL-22 which then acts on epithelial cells, further promoting tissue inflammation *via* alarmins such as the antimicrobial peptide HBD3 (161).

While many chronic inflammatory diseases are of unknown etiology, some have been correlated to dysbiosis of the skin microbiota. An example of this is atopic dermatitis (AD), a chronic T_h_2-dominated disease characterized by eczematous lesion and severe pruritus caused by immune cell infiltration of inflammatory DCs, macrophages and eosinophils (162). Further, AD is often found to be associated with transepidermal water loss due to a mutation in the filaggrin gene which leads to enhanced susceptibility to overgrowth of pathogenic *S. aureus* (163, 164). Further, patients with acute flares of the disease have been found to have an acute expansion of the cutaneous *S. aureus* population and significant loss of diversity in the cutaneous microbiome. Conversely, resolution of lesions has been association with a more diverse microbiome composition and contraction of the *S. aureus* population (165). Chronic inflammatory skin disorders still represent a major subset of disease with little mechanistic understanding of how T cell responses are shifted to cause disease.

#### Infection

It is becoming clear that the capacity of LCs in activating T cells in human skin is highly context dependent with their homeostatic role being more regulatory rather than activating T_eff_ cells. However, it has been demonstrated that LCs are indeed able to activate skin-resident T_em_ in the context of *C. albicans* infection, driving them to produce effector cytokines such as IFN-γ and IL-17 (81).

As the skin is constantly exposed to pathogens, the pool of T_rm_ in this and other organs is thought to reflect previous infections and exposures. In humans, many CD69^+^ T_rm_ have been shown to recognize prevalent viruses such as influenza A (166, 167), and respiratory syncytial virus (RSV) (168) in the lung. Further, viruses that cause latent and re-activating infections such as herpesvirus (HSV)-1 and -2 (72, 169, 170), Eppstein-Barr virus (171–173), and cytomegalovirus (174) are also known to elicit a strong T_rm_ response. This is further corroborated by the correlation between presence of virus-specific T_rm_ and increased immune protection and ability to control infections, which was shown to be the case for RSV (168), hepatitis B virus (175), and HSV-2 (170) infection. Specifically, in HSV infections, CD8^+^ T_rm_ seem to play a crucial role in resolution and protection. HSV-specific CD8^+^ T_rm_ have been found at the dermal-epidermal junction, close to sensory nerve endings which connect the latently infected ganglia to the skin as well as the genital mucosa (72, 170, 176). These cells have been shown to rapidly produce perforin and pro-inflammatory cytokines upon asymptomatic HSV-2 shedding. Further, cluster formation around virally infected epithelial cells and recruitment of CD8^+^ T cells from the dermis (170) emphasize that CD8^+^ T_rm_ are at the forefront of the immune response against acute and latent HSV. While it is now possible to also study T_rm_ in humans, it is worth mentioning that the great majority of current knowledge of T_rm_ behaviour during infection was acquired using murine models of HSV infection which greatly contributed to our understanding of these cells in mucosal tissues (11, 123, 177–180).

## Intestine

**Figure 4:**
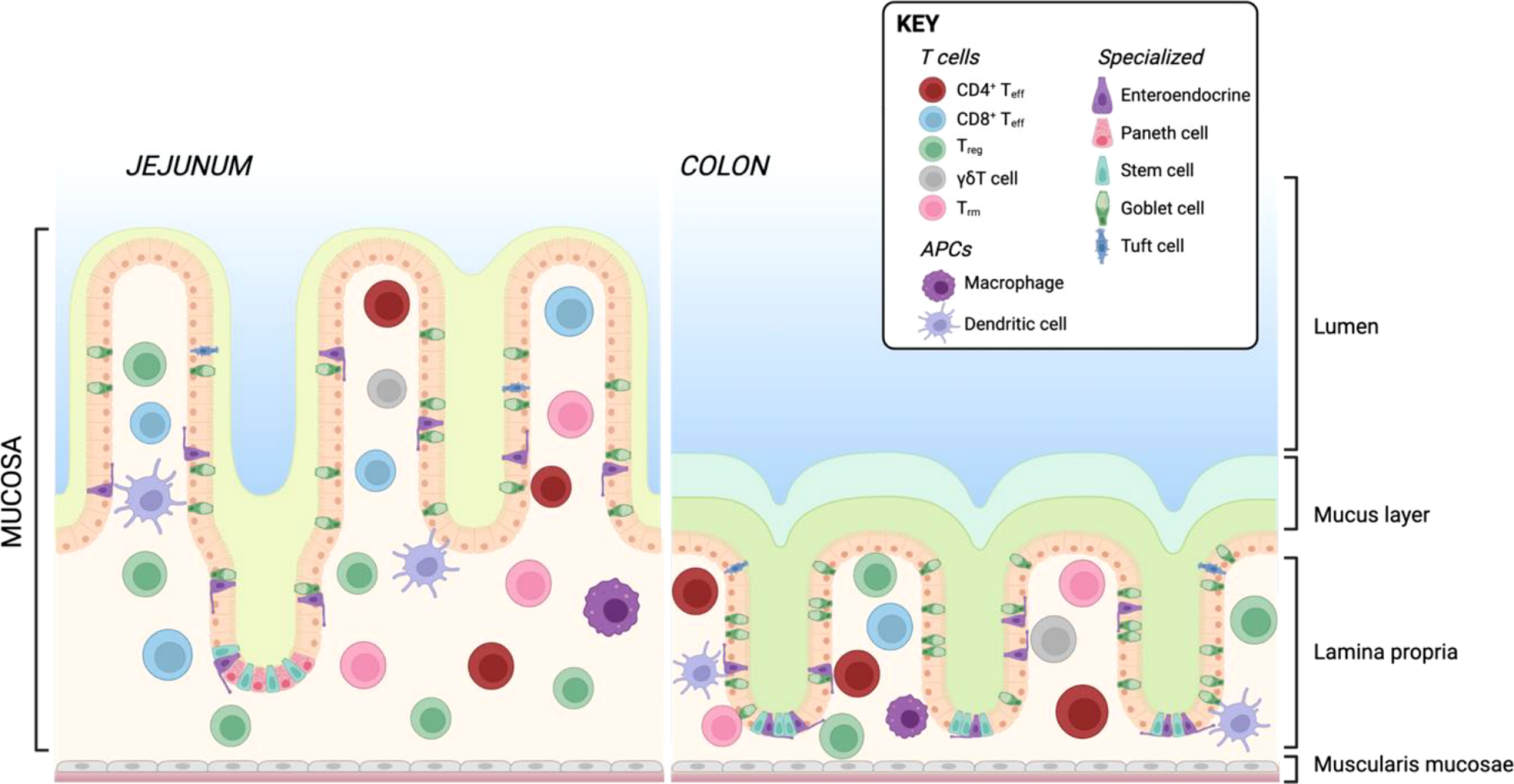
Resident T cells and APCs in the human small and large intestine. Created with BioRender.com.

Similar to the skin, the intestine is constantly exposed to exogenous triggers such as food or microbiota-derived antigens. These antigens are prevented from triggering a pathogenic immune response by cellular barriers. Physically, the intestine is protected by a layer of mucus and glycocalyx which coats the epithelial layer (181) and contains high concentration of secreted IgA (182, 183). In the small intestine, this is composed of a single unattached layer, while the large intestine has two layers of protective mucus, respectively relating to the bacterial burden in each location (184). The intestine is also home to intraepithelial lymphocytes (IELs), other immune cells resident in the lamina propria (LP) and gut-associated lymphoid tissue (GALT), comprising Payer’s patches (PP), cecal patches, and colonic patches distributed along the small and large intestine (185). There are differences in immune cell composition between the small and large intestine which have been extensively reviewed elsewhere (186, 187). A simplified overview of the architecture of the small and large intestine including resident T cells and APCs is shown in **Figure 4**.

At the bottom of the intestinal crypts, Paneth cells are the main producers of antimicrobial products such as defensins (188) and lysozyme (189), which are secreted into the mucus at the opening of the crypt. Goblet cells, responsible for the production of intestinal mucus, have the ability to take up antigen from the intestinal lumen and deliver these antigens to DCs in the LP *via* a process called goblet cell-associated antigen passage (GAP) (190). Antigens delivered *via* this process have been shown to be taken up by CD103^+^CD11c^+^ DCs which preferentially present to T_regs_, suggesting that this way of antigen delivery significantly contributes to induction of oral tolerance (191). While this mechanism is not well-understood yet, the more accepted route of antigen delivery from the lumen to the epithelium is *via* M cells on lymphoid follicles (e.g. on Payer’s Patches), which can transport whole bacteria (192, 193) that can then be taken up by DCs in the epithelium. This continued sampling of the microbiota by the immune system is crucial to maintaining homeostasis and resistance to pathogens. For example, expression of the chemokine receptor CX3CR1 in mice is essential for APCs to extend their dendrites between epithelial cells and take up intestinal bacteria from the lumen (194) which are then transported to the mesenteric LNs, where production of secretory IgA by plasma cells is induced (195–197). While originally being described as DCs due to their functional properties (194), CX3CR1+ APCs were classified as macrophages by others as they also express the macrophage markers CD64 and F4/80 and derive from monocytes (198, 199). Specifically, DCs in the intestine have the major responsibility in establishing tolerance to oral and microbiota-derived antigens. The gut-draining LNs as well as the GALT are the primary sites of T cell priming by intestinal DCs. As in other tissues, many DC subsets have been identified in the human intestine, with specific subsets more prevalent at specific anatomic locations. In humans, intestinal cDCs are divided into subgroups based on the expression of CD103 and SIRPα (200, 201), with CD103^-^SIRPα^+^ cDC2 further subcategorized based on the expression of the chemokine receptor CCR2 (202).

Intestinal cDCs are the only DCs expressing the enzyme RALDH2, which is required for metabolizing Vitamin A to all-trans retinoic acid (RA) (203). This metabolite is required for imprinting gut-homing receptors on T cells, namely α4β7 and CCR9 (204–207). Both CD103^+^ and CD103^-^ cDCs in humans have been found to express RALDH2 (208), which is reinforced by expression of RA by stromal cells in the mesenteric LNs (209, 210). In humans, the majority of IELs are T cells, with the highest proportion of non-T immune cells in the colon (211). The highest number of IELs are found in the proximal small intestine, decreasing in the distal small intestine, and lowest numbers in the colon (212). In the adult jejunum, the majority of IELs are CD8^+^ αβT cells with a tissue-resident T_em_ phenotype [reviewed in (213)], while the ileum and colon have higher numbers of CD4^+^ αβT cells, with a minor population of γδT cells (212). In the LP, CD4^+^ T cells dominate over CD8^+^ T cells, with the majority of cells exhibiting T_reg_-like or T_em_ phenotypes (214–217). IL-17 producing CD4^+^ T cells are most common in the LP of the colon and ileum, with lowest numbers in the jejunum (216), which is inverse to the distribution of T_reg_:non-T_reg_ T cells observed in mice (215, 217).

### DC-T cell composition in homeostasis

#### Memory T cells

In contrast to skin, sustained CD69 expression is not necessary for T_rm_ formation in the small intestine (7). Further, in the human intestine CD103 is also not necessary for T_rm_ persistence (218, 219), and is higher expressed on CD8^+^ T_rm_ than CD4^+^ T_rm_ (216, 220, 221). Human intestinal T_rm_ specifically express CD161, a C-type lectin^-^like receptor (222, 223), and they share the classic T_rm_ phenotype of downregulating LN homing receptors CD62L and CCR7 as well as the upregulation of adhesion molecules CRTAM and chemokine receptors CXCR6 and low expression of CX3CR1 (224). In the human small intestine, both CD4^+^ and CD8^+^ T_rm_ have been described to survive years, with CD4^+^ T_rm_ exhibiting a T_h_1 phenotype upon reactivation (218, 225). In the gut, it has yet to be elucidated if T_rm_ are continuously replenished from circulating T_cm_ under homeostatic conditions or whether the local population proliferates *in situ*, which has so far not been described. The TCR repertoire of CD8^+^ CD103^+^ vs. CD103^-^ T_rm_ has been described to have low clonal overlap, however overlap between CD103^-^ CD8^+^ T_rm_ was shown to be similar to that of T cells from the peripheral blood, indicating that CD103^-^ T_rm_ are recruited from the periphery and represent an intermediate state between circulatory and resident T cells (218). A study utilizing two-photon laser scanning microscopy revealed that intestinal T_rm_ have restricted mobility (226), indicating that intestinal T_rm_ are able to remain at the site of primary infection.

In mice, memory precursor cells expressing low levels of KLRG1 have been identified as a T_rm_ precursor, whose development is accelerated by DC-derived TGF-β (227). Inflammatory monocytes expressing IL-12 and TNF-β have been shown to suppress TGF-β-induced CD103 expression, leading to an increased population of CD103^-^ LP T_rm_ (228). Additionally, intraepithelial CD103^-^ T_rm_ appear to preferentially develop from KLRG1^+^ T cells over T cells that never express KLRG1 (229). Lastly, while IL-15 is critical for T_cm_ and T_em_ maintenance, this cytokine is not necessary for T_rm_ retention in the intestine (230).

Overall, T_rm_ biology and contribution of antigen presenting cells to T_rm_ generation and maintenance in the human intestine still have many open questions. More detailed reviews on intestinal T_rm_ can be found elsewhere (231, 232).

#### Effector T cells

While at steady-state, DCs in the gut preferentially induce T_regs_, with T_eff_ cells being primarily induced during infection or inflammation, which has mostly been studied in mice. Intestinal cDCs “escaping” regulatory conditioning in the gut at homeostasis have, however, been shown to induce tonic protective T_eff_ responses. This escape has been proposed to be mediated by early exposure to TLR ligands and pro-inflammatory cytokines, reducing residency time of cDCs and pDCs in the epithelium and thereby limiting exposure to regulatory-inducing factors (233, 234). Another example of this is p38-MAPK signaling in mouse CD103^+^ DCs, which has been shown to regulate fate-decision between T_reg_ and T_h_1 cells from infiltrating naïve T cells by influencing RALDH2 expression required for T_reg_ induction (235). Further, specific TLR5 signaling activating CD103^+^CD11b^+^ cDCs induces IL-6 and IL-23 production which promotes T_h_17 development and antimicrobial peptide production (200, 236).

The local microbiota is also essential in inducing T cell subset differentiation and polarization in the gut. In mice, it has been shown that monocolonization with segmented filamentous bacteria (SFB), which are members of the order Clostridiales, can induce the development of LP-resident CD4^+^ T_h_17 cells (237). This selective T_h_17 induction is MHC class II-dependent and requires presentation of SFB antigens by resident intestinal CD11c^+^ DCs (238). The relationship between SFB and T_h_17 has further been demonstrated in mice engineered to express the human antimicrobial peptide HBD5. These mice exhibited loss of SFB which subsequently correlated to a lower percentage of T_h_17 cells in the lamina propria (239).

#### Regulatory T cells

T_regs_ are central components of establishing tolerance in the intestine and crucial for maintaining homeostasis. Specifically in the gut, T_regs_ are necessary for controlling pro-inflammatory responses to commensal pathogens as well as establish tolerance to food antigens (240–242). Both thymus-derived (t)T_regs_ and periphery-induced (p)T_regs_ have been described in the gut, with pT_regs_ being thought to play the main role in establishing oral tolerance (243, 244), having been shown to control dysregulated T_h_1 responses to food antigens (245). In the colon, the predominant subset of pT_regs_ expresses the T_h_17 master transcription factor ROR-γt, the expression of which is dependent on the microbiota (245–248). The ROR-γt^-^ pT_regs_ conversely are critical for homeostasis maintenance in the small intestine (245). In mice, Helios^+^ tT_regs_ in the gut express GATA3 and exhibit a tissue-repair phenotype (246, 249, 250). This GATA3^+^ T_reg_ subset has not, however, been described in humans so far.

TGF-β is an essential cytokine for pT_reg_ differentiation and is, unsurprisingly, present at high concentrations in the intestine (251). DC-derived TGF-β in the gut is essential for local T_reg_ differentiation, which has been demonstrated in mice by ablating expression of the integrin responsible for activation of latent TGF-β (α_v_β_8_) on DCs which lead to impaired induction of T_regs_ in the mesenteric LNs (252). Contrarily, deletion of the TGF-βR1 on T_regs_ resulted in normal T_reg_ numbers in the gut (253). However, the authors did not analyze T_reg_ subsets in this study, therefore it cannot be excluded that compensatory T_reg_ expansion was the underlying cause for this observation. Other than cytokines, the metabolite RA is an important contributor to T_reg_ differentiation in the gut. Together with TGF-β, RA has been shown to induce pT_reg_ characterized by upregulation of CCR9 and α_4_β_7_ (254–256). Particularly CD103^+^ DCs are crucial for this induction, as they show a high expression of RALDH2, the enzyme metabolizing vitamin A to RA (257, 258). Particularly development of ROR-γt^+^ pT_regs_ is dependent on DC-derived RA (247, 259), further emphasizing that local T_reg_ induction is crucial to intestinal homeostasis. Other than RA, DCs play a role in T_reg_ induction *via* TLR signaling in the gut. For example, TLR2-mediated recognition of polysaccharide A on the commensal *Bacteroides fragilis* has been shown to trigger induction of T_regs_ and their production of the anti-inflammatory cytokine IL-10 (260).

#### γδT cells

Intestinal intraepithelial γδT cells play an extensive role in tissue surveillance, having a high migratory capacity and moving through the intestinal epithelium using occludin-mediated cell-cell contact (261). The majority of γδT cells in the human intestine express V7δ TCR (262) and have been associated with intestinal homeostasis *via* the production of keratinocyte growth factor 1 (KGF1) (263). Their significant contribution to gut homeostasis has been shown in γδT cell deficient mice, showing that mice lacking these cells have reduced intestinal epithelial cell turnover (264), increased susceptibility to dextran sulfate sodium (DSS)-induced colitis (263), and increased gut permeability (265). In humans, intestinal γδT expressing NKG2A have been shown to express TGF-β1, thereby dampening IFN-γ and granzyme B production by co-cultured αβT cells from patients with coeliac disease (266). Together, studies so far indicate that intestinal γδT cells have an important role in regulating tissue homeostasis and contribute to controlling inflammatory responses in the gut. However, a lot of open questions about their effector functions and interplay with other cells, such as APCs, in humans still remain.

### DC-T cell composition in inflammation and infection

#### Chronic inflammatory diseases

Inflammatory bowel disease (IBD) is a well-known and well-studied chronic inflammatory condition in the intestine and covers ulcerative colitis and Crohn’s disease. IBDs have been linked with multiple exogenous factors such as environmental factors, microbiota dysbiosis, and genetic background (267, 268), which culminate in an overall inappropriate immune cell activation in the gut. In IBD, DCs are known to contribute to disease pathology *via* TLR2/4-induced production of IL-12, IL-6, and IL-23 (269, 270), which further impacts T cell polarization and drives T_h_17-mediated disease phenotypes. CD103^+^CD141^+^CD1c^+^ cDCs are reduced in inflamed intestinal lesions, showing functional impairments such as decreased RALDH2 activity (271). Further, some findings have indicated that intestinal inflammation, such as seen in Crohn’s disease, impairs normal DC trafficking which consequently leads to dysregulated T cell responses in the gut. For example, CCR7 expression on CD83^+^DC-SIGN^+^ intestinal cDCs is lower in patients with Crohn’s disease (272). Further, it has been observed that leptin production in mesenteric fat is increased in early Crohn’s disease patients (273), which has been associated with upregulation of CCR7, maturation and migration of cDCs (274). Whether CCR7 expression is timepoint dependent and what effect this has on T cell priming in Crohn’s disease remains to be elucidated.

In recent years, the role of T_rm_ in IBD has become apparent. For example, CD69^+^CD103^+^ T_rm_-like cells in the LP have been described to be increased in patients with ulcerative colitis and Crohn’s disease. Further, the authors could show that increased levels of CD4^+^ T_rm_ are associated with early IBD relapse (275). Along the same line, Bishu et al. described these CD4^+^ T_rm_ as functionally competent TNF-α producers in inflamed tissue of patients with Crohn’s disease (276). CD8^+^ T_rm_ have also been implicated in IBD pathogenesis. Bottois et al. described two distinct subsets of CD8^+^ T_rm_ expressing KLRG1 and CD103, showing that CD103^+^ CD8^+^ T_rm_ in Crohn’s disease patients exhibit a T_h_17-like phenotype, while highly proliferative KLRG1^+^ CD8^+^ T_rm_ present with increased cytotoxic effector function and are overrepresented during acute inflammation (277). Single-cell RNA-sequencing studies of ulcerative colitis also showed transcriptional changes in the CD8^+^ T_rm_ compartment, with an increased inflammatory signature (278, 279). In a recent publication using mass spectrometry, HLA-DR^+^CD38^+^ CD4^+^ T_em_ were found to be enriched in lesions of Crohn’s disease patients. The authors could further use imaging mass cytometry of tissue sections to show co-localization of memory CD4^+^ T cells together with HLA-DR^+^CD11c^+^ DCs located below the epithelial layer in the inflamed regions of the intestine (280). T_rm_ with a regulatory signature have also been described to be reduced in IBD, characterized by CD103^+^Runx3^+^ and expression of the regulatory-associated molecules CD39 and CD73 together with IL-10 production (281). Furthermore, studies revealed a decrease in both the CD103^+^ CD8^+^ and CD4^+^ T_rm_ compartment during active IBD, which recovered during remission phases, whereas the opposite observation was made for CD103^-^ T_rm_ (282). These studies further demonstrate the heterogeneity of intestinal T_rm_ and are likely a reflection of T_rm_ plasticity during different phases of the inflammatory response.

#### Infection

While the physical barriers like the intestinal mucus protect against food-borne pathogens and harmful commensals (known as pathobionts), many microbial organisms have evolved to evade host defense and cause infections. Infections with such enteric pathogens are most commonly associated with diarrhea, which is a major cause of death worldwide (283). The most frequent enteric infections are with *Salmonella* spp. and *Campylobacter* spp (284)., with other examples being *Vibrio cholerae*, *Shigella* spp. and certain strains of *Escherichia coli* (285). The most common pathobiont infections are caused by *Enterococcus* spp (286). and *Clostridium difficile* (287).

While TLR2 is important in inducing T_regs_ (see above), TLR5, the receptor for bacterial flagellin (288), has been implicated in the host response to invasive pathogens such as *Salmonella* spp. CD11c^+^ LP-resident DCs express TLR5, which is important in modulating DC movement, as TLR5-deficient mice have increased survival and lower dissemination when infected orally with *Salmonella* spp. whereas no difference was observed when mice were infected intraperitoneally (289). This observation indicates that trafficking to the mesenteric LN by DCs is impaired thereby preventing dissemination of the infection. Another important consequence of TLR-mediated activation of DCs is cytokine production. A crucial cytokine in the gut produced by DCs in response to infection is IL-23, which has been linked to infection with pathogens like *Salmonella* spp (290)., *C. rodentium* (291), and *C. jejuni* (292). The receptor for IL-23 in the gut is expressed on multiple immune cells such as T_h_17, NKT, γδT cells and ILCs (293, 294). IL-23 receptor signaling in turn triggers production of IL-17 and IL-22. IL-17 appears to have time-dependent effects during intestinal infection. During early *Salmonella* spp. infection, IL-17 produced in the caecum is primarily mediated by T_h_17 cells and to a lesser extent γδT and NKT cells (295, 296). Another example of the importance of T_h_17-mediated immunity during infection has been shown in rhesus macaques where SIV-induced depletion of T_h_17 cells leads to erosion of the mucosal barrier and increased dissemination of *S*. *enterica* Typhimurium to the mesenteric LNs (296). IL-17A or IL-17F deficiency in mice lead to increased pathology in response to *C. rodentium* infection (297).

The IL-23-T_h_17 axis is also important in human intestinal infection. Patients suffering from *C. jejuni* infection show increased percentages of T_h_1 and T_h_17 cells, as well as increased levels of the respective effector cytokines. The authors could show that when intestinal epithelial cells were treated with IL-17A or IL-17F, intracellular survival of *C. jejuni* was significantly decreased, emphasizing the importance of these cytokines in human infection (292). Further, IL-17 expression was also detected in the duodenum of patients recovering from *V. cholerae* infection, the causative agent of cholera. Kuchta et al. observed that in patients suffering from acute cholera, IL-17 expression was increased compared to later disease stages or healthy subjects, suggesting that *V. cholerae* infection also induces an immediate mucosal T_h_17 response (298).

The other IL-23-induced cytokine important in intestinal infection is IL-22. In general, IL-22 is associated with tissue repair and is known to be a major inducer of antimicrobial peptide production by mucosal epithelial cells (299, 300). In the context of infection, IL-22 has been found to increase colonization resistance to the pathobiont vancomycin-resistant enterococci (236). Similar to IL-17, IL-22 has also been shown to have time-dependent effects. During early infections, IL-22 is primarily produced by ILCs and only later on by T cells. This was demonstrated by Ahlfors et al, showing that during infection with *C. rodentium* IL-22 is initially produced by ILC3s and then by CD4^+^ T cells (301).

Overall, it has become clear that the DC-induced IL-23-T_h_17 axis is particularly important in response to intestinal infection by modulating epithelial microbial peptide expression and preventing dissemination of intestinal infection.

## Female reproductive tract

The immune system in the FRT has a dual role as it protects the barrier tissue against pathogens transmitted during sexual intercourse, and promotes tolerance to foreign antigens necessary to allow fertilization and embryo development. As these two diametrical roles are important at specific times during the menstrual cycle, the composition of immune cells undergoes major fluctuations. During menstruation, a much higher density of CD1a^+^ DCs was observed in the human uterus compared to proliferative and secretory phase (302). Uterine macrophages increase constantly in numbers during secretory phase and peak at menstruation, while the total number of T cells remains constant (303–305). The sex hormone progesterone does not only inhibit activation of DCs (306), but also causes polarization of T cells into T_h_2 and T_reg_ direction (307, 308). Moreover, subsets of immune cells do not only change during the menstrual cycle, but also differ when comparing tissues from pre- and postmenopausal females (309). There are substantial differences between the structure and physiology of the female genital tract between the most frequently used animal model of mice compared to humans, as the murine uterus contains two uterine horns and also the estrous cycle has a length of around 5 days compared to 28 days in humans. However, due to the previously low interest in female reproductive health, scientists started only recently to investigate immune cell populations in large scale in the FRT of humans. Therefore, most knowledge on the female genital immune system was obtained in mice (310). With this section, we aim to shed light on specific features of antigen uptake and presentation as well as T cell responses in the female genital tract and raise awareness for inflammatory conditions and chronic infections.

**Figure 5:**
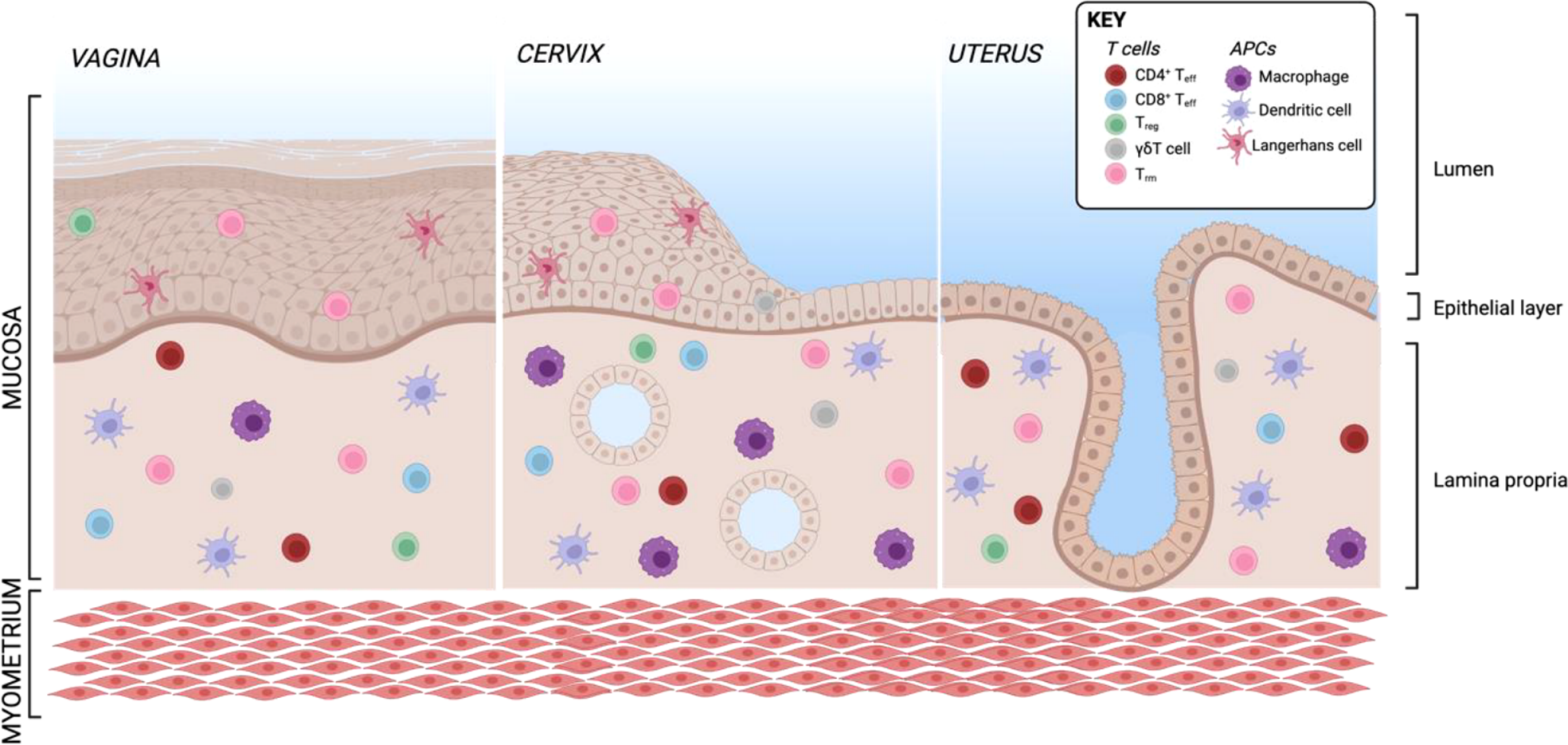
Resident T cells and APCs in the human FRT. Created with BioRender.com.

The female genital tract is structured in several parts: the lower reproductive tract lined with multilayered stratified epithelia forming vagina and ectocervix, the endocervix as an interphase and the upper genital tract with single columnar epithelium forming the uterus, adjacent to the fallopian tubes stretch connecting the ovaries with the uterus which are composed of secretory and ciliated columnar epithelial cells (**Figure 5**). The main APC subsets in the human vaginal tissue are, similar to skin, in the epithelial layer LCs characterized by CD207 expression and in the lamina propria DCs characterized by expression of CD1c as well as CD14 on a specific subset (311, 312). In addition to DCs, another frequent APC subsets in the vagina are CD1c^-^CD14^+^ macrophages additionally having CD163 on their surface (311, 312). In the cervix, the most frequent immune cell population are macrophages which make up more than 25% of all CD45^+^ immune cells (55). CD11c^+^CD14^+^ DCs accounting for another approx. 20% of immune cells are the most common DC subset and a large proportion also express DC-SIGN. Other DC subsets such as CD11c^+^CD14^-^ myeloid DCs and CD123^+^ plasmacytoid DCs were described in low numbers (55). The percentage of APC subsets within CD45^+^ immune cells is quite similar in cervix and uterus, however, the APC compartment in the uterine endometrium shows some substantial differences. There are less DC-SIGN^+^ DCs and DCs expressing CD103^+^ involved in antigen sampling and migration were almost exclusively found in the endometrium (313). In the murine endometrium, both CD103^+^ and CD103^-^ DCs migrate to the local lymph nodes upon antigen challenge. The CD103^+^ DCs preferentially present antigens to T_regs_, whereas their CD103^-^ counterparts were shown to stimulate an effective CD4 T cells response (314). In the murine uterus, DC in the decidua of pregnant females were shown to be trapped in the tissue, despite keeping responsiveness to pro-inflammatory stimuli and migration capacity towards CCL21 (315). This indicates that by preventing DC trafficking to the draining LN, T cell tolerance to fetal antigens is promoted. Single-cell sequencing of human uterine samples during secretory and proliferative phase revealed presence of myeloid cells during both phases, being composed of DCs as well as M1- and M2-polarized macrophages (316). CD11c^+^ DCs can be further divided into CD11b^++^ and CD11b^lo^ DCs, with the CD11b^+^ expressing DCs being the most abundant subset in all tissues of the FRT and correlating with CD14 expression (313). In the vagina, the ratio of CD4 to CD8 cells is almost equal, with an increasing ratio towards endocervix and ectocervix (55, 317). In the uterus, however, CD8 T cells represent the predominant subset (55). B and NK cells make up less than 5% of immune cells in the human ectocervix and are not in focus of this review (55).

### DC-T cell composition in homeostasis

#### Memory T cells

In general, most T cells in the female reproductive tract are T_rm_ being CCR7^-^CD45RA^-^. More than 80% of cervical T cells express CD69 within both stroma and epithelium (317, 318). The marker CD103 being associated with a T_rm_ phenotype in other tissues is in the cervix almost exclusively present on epithelial CD8 T_rm_ (318), but also enriched on vaginal CD4 T_rm_ (317). These vaginal CD103^+^CD69^+^ CD4 T_rm_ show a T_h_17 signature including high expression of RORC, IL-17A, IL-17F and IL-22 (317). A recent publication used T_rm_ derived from human cervix to assess antigen-specific CD4 and CD8 response against HSV-2 (319). An elegant mouse study using parabiosis models revealed that CD8+ T_rm_ in the mucosa undergo proliferation *in situ* after mucosal rechallenge independently of CD11c+ DCs (114). On the other hand, bystander memory CD8 T cells consisting of T_cm_ and T_em_ are recruited during local challenge without antigen recognition and develop a T_rm_-like phenotype by upregulating CD69, but not CD103 (114). To investigate how the recruitment of bystander memory cells to sites of infection as well as tissue autonomous amplification of local T_rm_ contributes to immunity in the human FRT, it is important to apply functional models with human cells and validate other experimental approaches in the future. To date, the T_rm_ subset is the best studied immune cell subset in the FRT and will be discussed further in the sections about the respective infectious diseases.

#### Regulatory T cells

Recently, T_regs_ were shown to make up around 15% of the CD4 population with comparable percentage within all tissues from the lower FRT, including vagina, endocervix and ectocervix (317, 320). It is reported that T_regs_ are induced in the decidua of mice and humans to protect the developing embryo from the immune system of the mother, nicely summarized in the following reviews (321–323). However, T_regs_ can also have an unfavorable role if they dampen the immune response against sexually transmitted infections such as human immunodeficiency virus (HIV), human papilloma virus (HPV) or chlamydia. In a mouse model of intravaginal *N. gonorrhea* infection TGF-β^+^ T_regs_ were induced in cervix-draining lymph nodes, thus evading the immune response and enabling pathogen survival (324). The occurrence of T_regs_ in the mucosal tissue is described for several pathogens and conditions, while the mechanisms of their induction still need to be elucidated.

#### γδT cells

Human studies revealed a γδT cell percentage ranging from 5% to 10% of CD3^+^ T cells depending on tissue sampling during the proliferative phase or secretory phase. The majority of them expressing Vδ1 (325, 326), but CCR5 can be found on the surface of both Vδ1 and Vδ2 (327). HIV infection significantly reduces the number of γδT cells in the cervix (327). Abnormal vaginal flora due to bacterial vaginosis was shown to change the composition of vaginal γδT cells to higher levels of Vδ2 (328). Beside their role during infection, γδT cells seem to be involved in tolerance induction during pregnancy. The decidua of women with spontaneous abortions showed increased numbers of γδT cells with an additional upregulation of Vδ2^+^ cells (325). In the murine female genital tract, γδT cells represent a much higher proportion of immune cells and express preferentially IL-17A under steady state (329). As IL-17A was described to be essential for resistance against fungal infection, a murine study revealed that TCRγδ deficient mice are more susceptible to *C. albicans* growth in the FRT (330). To date, our knowledge about γδT cells in the FRT is still limited and remains to be addressed in different disease settings.

### DC-T cell composition in infection

#### Viral infections

CD4 and the chemokine receptors CCR5 and CXCR4 are hijacked by HIV. Beside T cells, this repertoire of receptors is found on all four APC subsets in the vagina in different quantities, indicating a role of these cells during HIV acquisition and transmission to other cell types (311). It was shown that exclusively CD14^+^ DCs take up HIV virus-like particles and express CCR5 ligands (313). The type-I interferon inducible lectin Siglec-1 expressed on CD14^+^ DCs was identified to play an indispensable role in HIV uptake and transmission to CD4 T cells which can be blocked by anti-Siglec-1 antibodies (331). As CD14^+^ DCs are most frequently occurring in the ectocervix, this tissue is highly relevant to study HIV transmission (313, 332). CD4 T_rm_ from the ectocervical region expressing CD69 are characterized by high CCR5, thereby function as a primary target for HIV infection and persistence (333, 334). Numbers of CD4 T_rm_ are significantly decreased in cervix tissue of infected individuals, but increased activation can be observed (333). In the same lines, CXCR3^+^ T_rm_ in the skin and anal mucosa of HIV infected individuals starting antiretroviral therapy late remain constantly depleted, thereby creating an optimal environment for HPV related cancer development (335). HIV-infected individuals show increased T_regs_ and reduced T_h_17 cells, the ratio between these two cell types can be restored by anti-retroviral therapy (ART) (336). The percentage of T_regs_ remained increased even under ART and was associated with a skewed ratio of CCL17/CCL20 in the ectocervix samples of these women (336), indicating that APCs as major source of those cytokines, are causing the disbalance of T cells in these conditions.

Infections with HPV are widespread and almost every human encounters HPV during their life time. There are several different types, with only some of them being transmitted sexually and causing infections that can lead to cancer development in the cervix. Patients with HIV infection possess an increased risk to develop HPV associated cancer with T cells as important players in the course of HPV-related malignancies (337, 338). Upon HPV infection, T cells in the cervix obtain a more activated profile by upregulation of HLA-DR, independent of HIV status of the patients (339). However, in patients with a co-infection of HIV and HPV, lower numbers of CD4 T cells were observed compared to HPV-negative HIV-infected patients (339). In individuals with HPV-associated genital warts, an accumulation of T_regs_ was reported (340). It was shown that T_regs_ are attracted by CCL17 and CCL22, which are mainly produced by CD1a^+^ LCs and macrophages within the warts, respectively (340). Trafficking of APCs such as LCs is impaired in HPV lesions, as the chemoattractant for (CCL20) and activation pattern of LCs (CCR7, CD80 and CD86) seem to be decreased (341–343). Also, T_h_17 cells seem to play a role in progression of HPV-related intraepithelial cervical neoplasia (CIN), as patients with high CIN or cervical cancer exhibit high numbers of T_h_17 cell in the blood, which is correlated with high IL-17 levels in the cervix tissue (344). In a study assessing the T cell infiltration in cervical cancer patients, CD103^+^ CD8 T cells infiltrate the tumors and are associated with good prognosis (345). These findings indicate that a T_h_17 and T_reg_ response is correlated with progressive HPV infection, whereas CD8 T cells are beneficial. However, most studies focus on late stages in CIN progression/tumor development and little is known about early processes of HPV infection.

#### Bacterial infections

Infections with chlamydia are the most common bacterial sexually transmitted infection in humans. However, most of our knowledge of immune reactions during chlamydia infections was obtained in mice, as studying immunity against *chlamydia trachomatis* (Ct) is connected with many difficulties, such as the high number of asymptomatic cases and the development of tolerance instead of immunity when using inactivated bacteria. The later problem was addressed in a mouse model by Stary et al. showing that live and UV-inactivated Ct are taken up by either CD103^-^ and CD103^+^ DC subsets, causing priming of immunogenic effector T cells or T_regs_, respectively (314). In mice, induction of T_h_1 cells plays a huge role in conveying protective immunity, whereas stimulation of CD8^+^ T cells was suggested to play a role in chronical inflammation and cause tissue destruction rather than advancing protective immunity in mice (314, 346, 347). T_h_1 polarization initially relies on IL-12 production by DCs, as IL-12 deficient mice had prolonged times of chlamydia shedding (348). In fact, the most important immune mechanism for chlamydia clearance is IFN-γ, as T-bet deficient mice could not control *chlamydia* growth, but T cells shifted to a more T_h_17 response, whereas IFN-γ or IFN-γ-receptor deficient mice die from systemic infection (349, 350). T_rm_ of the FRT seemed to be essential to protect against subsequent chlamydia infection (314). However, a recent publication suggests that also circulating memory T cells can protect against infection without being primed in the tissue (351). Apart from conveying protective immunity, T cells can also be involved in undesirable responses causing FRT pathology and chronic inflammation. Especially activation of non-antigen-specific CD4 as well as CD8 bystander cells can exacerbate the pathology in a mouse model of chlamydia infection (352). The presence of T_regs_ was on the one hand shown to exacerbate Ct infection (314), on the other hand, they are described to skew T cell differentiation into a T_h_17 direction, which was correlated with increased pathology in a *chlamydia muridarum* mouse model of infection (353). Together, these findings suggest that the T cell response during Ct infection is highly plastic and the induction of a certain cytokine milieu is essential.

## Discussion

### All the same: Commonalities and differences in tissue APC-T cell crosstalk

When comparing the three different tissues summarized in this review, some overarching themes are apparent: The majority of T cells in tissues are T_rm_ cells (7), closely followed by T_regs_ (19, 20), both cell types reflecting the constant exposure to environmental compounds and antigens in barrier tissues and the need for a balance between immune tolerance and reaction. Further, DC subsets are responsible for controlling this balance, but they are often described by different markers in different tissues and their subsets appear more tissue-specific than those of T cells, whose identity is often easier to define across tissues. However, some clear differences exist also in T cells. Expression of CD69 and CD103, canonical T_rm_ markers in the skin (13, 111) and FRT (317, 318), seem dispensable for T_rm_ establishment in the intestine (7). T_rm_ are relatively stationary within the respective tissue, however, there are quite substantial differences in motility between T_rm_ in different tissues, as T_rm_ in the FRT move up to 5-times faster compared to T_rm_ in skin epidermis, probably depending on the architecture of the tissue and density of the structural cells (114). While CD4^+^ and CD8^+^ T_rm_ exist in all discussed barrier tissues, the skin harbors more CD8^+^ T_rm_ than the intestine and the FRT, where the distribution of CD4^+^:CD8^+^ T_rm_ is approximately equal (218, 225). Further, T_reg_ induction in the intestine is highly dependent on RA produced by local DCs (254–256) and in the FRT, progesterone (307), independently of DCs, appears to take a similar role, while no hormones or metabolites are yet identified to induce T_regs_ in the skin. In general, it appears, that while all barrier tissues are continuously exposed to microbial antigens, only the intestine has dedicated DC subsets to specifically induce T_regs_ to promote tolerance against the microbiome (191). This observation fits with the fact that, in the skin, most T_regs_ respond and get activated by non-antigenic stimuli while most T_regs_ (127) in the gut are antigen specific (240–242). In general, aside from their function in maintaining immune tolerance, the function of T_regs_ in different tissues is often diverse, ranging from direct suppression of activated immune cells to aiding in tissue repair (19, 20), thereby emphasizing the need to characterize these cells and their non-canonical functions in a tissue context better. Similar to this, γδT cells exhibit both regulatory and cytotoxic functions across tissues even though their distribution is tissue-specific (Vδ1 in the skin, Vδ7 in the intestine, Vδ1 and Vδ2 in the FRT) (149).

During an immune challenge in barrier organs, such as during infection, T_rm_ are poised locally in all three tissues, reacting to previously encountered antigenic stimuli directly. Further immune responses are induced by APCs which traffic to the respective draining lymph nodes and recruit T_eff_ cells to the tissue. T_h_17 responses are crucial in controlling infections, both bacterial and viral (313, 332). Interestingly, the same responses and effector cytokines are also often the ones that are pathogenic in chronic inflammatory diseases (153, 154). How and why exactly these exacerbated immune responses cannot be controlled by tissue-resident T_regs_, which are present in barrier tissues in great abundance under homeostatic conditions, has yet to be elucidated. However, all chronic inflammatory diseases discussed in this review are characterized by a decrease in tissue T_regs_, but whether this is cause or effect of chronic tissue inflammation and what role APCs play in this shift of T cell subsets during chronic inflammation remains a big question that should be the topic of further research.

### Into the (un)known: On big data, future perspectives, and individualized therapies

Previous dogmas of dividing immune responses strictly into pro- and anti-inflammatory immune cell subsets are outdated. The more we learn about tissue-specific immune responses, the more we understand that there is not the one beneficial and harmful immune cell subset to every disease. It is more a fine-tuned balance act between APCs and T cells to enable immunity against pathogens but protect the host from autoimmunity. With current advances in single-cell RNA sequencing (scRNA-seq) and multichannel flow cytometry, we will be able to get a better insight, which players are involved in regulating immunity during homeostasis. scRNA-seq has specifically enabled much greater insight into molecular mechanisms of tissue immunity as well as led to the discovery of new immune cell subsets or new definitions of existing subsets. This is especially valuable since this approach allows for the acquisition of a large amount of data from, often limited, human material. Further, a lot of information that is derived from these big data experiments would be impossible to acquire using traditional experimental models as it is now possible to also model *in vivo* dynamics from these datasets, such as the interplay between different cell types (354, 355) and temporal dynamics across the development of organs (356–358), and tracking T cell clones across tissues (359, 360). Analyses like these have revealed novel regulatory T cell-APC interactions at the maternal-fetal interface important for embryo implantation (361), a renewed focus on pDCs in skin inflammation (362), novel Vδ1 T cell effector subsets (363), and detailed profiling of different immune niches and interactions across the human intestine (54). Further, a better understanding of tissue adaptation of different immune cells is becoming appreciated, highlighting basic principles of immune biology in barrier tissues but also appreciating that these cells have the potential to specifically adapt to the local tissue environment and how this changes in disease (126, 364–366). As highlighted in this review, communication between different immune cell types is absolutely essential in determining the outcome of an immune response and understanding this interplay at a deeper level in local tissues is an important step towards developing new therapeutic avenues that can act in a much more targeted manner than previously possible. Further, the plasticity of immune cell subtypes, especially APCs and T cells, is becoming more appreciated as having whole transcriptome data can separate cell types that were previously indistinguishable and is an important step towards understanding fundamental changes during disease development. As this knowledge progresses, it will be interesting to see if we will gain a better understanding of responses to immunotherapy and why some patients benefit while others do not. Moreover, this technical evolution will also allow to come away from animal models and help uncover tissue-specific differences as well as overarching themes in immune defense in barrier tissues. In addition, we want to emphasize the importance of investigating the interplay of different human immune cell subtypes in complex 3D model systems to further validate findings from big data-based models and how these can be translated to patient care. It will be crucial to define the function of rare DC subsets, T_regs_ or γδT cells as they seem to have a major role in immune balance despite their low frequencies. Especially the mechanisms balancing different γδT cell subset or T_regs_ and T_h_17 cells will be an important focus for further studies. In the future, integrating different large datasets will be highly valuable in better understanding more complex disease systems, such as metabolic dysregulation as well as epigenetic modifications. Together, these data will yield a clearer picture of biological networks and how they are perturbed in different diseases. Currently, we are at the start of a new era of understanding biological mechanisms that lead to disease and disease progression. In the future, insights gained from these basic studies will in turn re-shape how therapeutics are developed and most likely emphasize the importance of more patient-specific approaches to health care.

## Author contributions

All authors listed have made a substantial, direct, and intellectual contribution to the work and approved it for publication.

## Funding

Funding was received from the Austrian Science Fund (P31494) and the Austrian National Bank (17872), which include publication costs.

## Conflict of interest

The authors declare that the research was conducted in the absence of any commercial or financial relationships that could be construed as a potential conflict of interest.

## Publisher’s note

All claims expressed in this article are solely those of the authors and do not necessarily represent those of their affiliated organizations, or those of the publisher, the editors and the reviewers. Any product that may be evaluated in this article, or claim that may be made by its manufacturer, is not guaranteed or endorsed by the publisher.

## References

[1] Sowell RT Rogozinska M Nelson CE Vezys V Marzo AL. Cutting edge: Generation of effector cells that localize to mucosal tissues and form resident memory CD8 T cells is controlled by mTOR. J Immunol (2014) 193:2067–71. doi: 10.4049/jimmunol.1400074 PMC4134982 25070853

[2] Gaide O Emerson R Jiang X Gulati N Nizza S. Common clonal origin of central and resident memory T cells following skin immunization. Nature (2015) 21:647–53. doi: 10.1038/nm.3860 PMC4632197 25962122

[3] Laidlaw B Zhang N Marshall H Staron M Guan T. CD4+ T cell help guides formation of CD103+ lung-resident memory CD8+ T cells during influenza viral infection. Immunity (2014) 41:633–45. doi: 10.1016/j.immuni.2014.09.007 PMC4324721 25308332

[4] Wakim LM Woodward-Davis A Liu R Hu Y Villadangos J Smyth G. The molecular signature of tissue resident memory CD8 T cells isolated from the brain. J Immunol (2012) 189:3462–71. doi: 10.4049/jimmunol.1201305 PMC3884813 22922816

[5] Zhou X Yu S Zhao D Harty J Badovinac V Xue H. Differentiation and persistence of memory CD8+ T cells depend on T cell factor 1. Immunity (2010) 33:229–40. doi: 10.1016/j.immuni.2010.08.002 PMC2928475 20727791

[6] Intlekofer A Takemoto N Wherry E Longworth S Northrup J Palanivel V. Effector and memory CD8+ T cell fate coupled by T-bet and eomesodermin. Nat Immunol (2005) 6:1236–44. doi: 10.1038/ni1268 16273099

[7] Steinert EM Schenkel JM Fraser KA Beura LK Manlove LS Igyártó BZ. Quantifying memory CD8 T cells reveals regionalization of immunosurveillance. Cell (2015) 161:737–49. doi: 10.1016/J.CELL.2015.03.031 PMC4426972 25957682

[8] Sallusto F Lenig D Förster R Lipp M Lanzavecchia A. Two subsets of memory T lymphocytes with distinct homing potentials and effector functions. Nature (1999) 401:708–12. doi: 10.1038/44385 10537110

[9] Masopust D Vezys V Marzo AL Lefrançois L. Preferential localization of effector memory cells in nonlymphoid tissue. J Immunol (2014) 192:845–9. doi: 10.1126/SCIENCE.1058867 24443507

[10] Reinhardt R Khoruts A Merica R Zell T Jenkins M. Visualizing the generation of memory CD4 T cells in the whole body. Nature (2001) 410:101–5. doi: 10.1038/35065111 11242050

[11] Gebhardt T Wakim L Eidsmo L Reading P Heath W Carbone F. Memory T cells in nonlymphoid tissue that provide enhanced local immunity during infection with herpes simplex virus. Nat Immunol (2009) 10:524–30. doi: 10.1038/ni.1718 19305395

[12] Masopust D Choo D Vezys V Wherry E Duraiswamy J Akondy R. Dynamic T cell migration program provides resident memory within intestinal epithelium. J Exp Med (2010) 207:553–64. 10.1084/jem.20090858 PMC2839151 20156972

[13] Jiang X Clark RA Liu L Wagers AJ Fuhlbrigge RC Kupper TS. Skin infection generates non-migratory memory CD8+ TRM cells providing global skin immunity. Nature (2012) 483:227–31. doi: 10.1038/nature10851 PMC3437663 22388819

[14] Schenkel JM Fraser KA Vezys V Masopust D. Sensing and alarm function of resident memory CD8+ T cells. Nat Immunol (2013) 14:509. doi: 10.1038/NI.2568 23542740 PMC3631432

[15] Iijima N Iwasaki A. A local macrophage chemokine network sustains protective tissue-resident memory CD4 T cells. Sci (1979) (2014) 346:93–8. doi: 10.1126/SCIENCE.1257530 PMC4254703 25170048

[16] Glennie N Yeramilli V Beiting D Volk S Weaver C Scott P. Skin-resident memory CD4+ T cells enhance protection against leishmania major infection. J Exp Med (2015) 212:1405–14. doi: 10.1084/jem.20142101 PMC4548053 26216123

[17] Clark RA Chong B Mirchandani N Brinster NK Yamanaka K Dowgiert RK. The vast majority of CLA+T cells are resident in normal skin. J Immunol (2006) 176:4431. doi: 10.4049/jimmunol.176.7.4431 16547281

[18] Gebhardt T Palendira U Tscharke DC Bedoui S. Tissue-resident memory T cells in tissue homeostasis, persistent infection, and cancer surveillance. Immunol Rev (2018) 283:54–76. doi: 10.1111/IMR.12650 29664571

[19] Burzyn D Benoist C Mathis D. Regulatory T cells in nonlymphoid tissues. Nat Immunol (2013) 14:1007–13. doi: 10.1038/ni.2683 PMC4708287 24048122

[20] Shao Q Gu J Zhou J Wang Q Li X Deng Z. Tissue tregs and maintenance of tissue homeostasis. Front Cell Dev Biol (2021) 9:717903. doi: 10.3389/FCELL.2021.717903 34490267 PMC8418123

[21] Kim J Rasmussen J Rudensky A. Regulatory T cells prevent catastrophic autoimmunity throughout the lifespan of mice. Nat Immunol (2006) 8:191–7. doi: 10.1038/ni1428 17136045

[22] Sakaguchi S Yamaguchi T Nomura T Ono M. Regulatory T cells and immune tolerance. Cell (2008) 133:775–87. doi: 10.1016/J.CELL.2008.05.009 18510923

[23] Smigiel KS Richards E Srivastava S Thomas KR Dudda JC Klonowski KD. CCR7 provides localized access to IL-2 and defines homeostatically distinct regulatory T cell subsets. J Exp Med (2014) 211:121–36. doi: 10.1084/JEM.20131142 PMC3892972 24378538

[24] Huehn J Siegmund K Hamann A. Migration rules: Functional properties of naive and effector/memory-like regulatory T cell subsets. Curr Top Microbiol Immunol (2005) 293:89–114. doi: 10.1007/3-540-27702-1_5 15981477

[25] Cretney E Kallies A Nutt S. Differentiation and function of Foxp3+ effector regulatory T cells. Trends Immunol (2013) 34:74–80. doi: 10.1016/j.it.2012.11.002 23219401

[26] Konijnenburg Dv Reis B Pedicord V Farache J Victora G Mucida D. Intestinal epithelial and intraepithelial T cell crosstalk mediates a dynamic response to infection. Cell (2017) 171:783–94. doi: 10.1016/j.cell.2017.08.046 PMC5670000 28942917

[27] McGinley A Edwards S Raverdeau M Mills K. Th17 cells, γδ T cells and their interplay in EAE and multiple sclerosis. J Autoimmun (2018) 87:97–108. doi: 10.1016/j.jaut.2018.01.001 29395738

[28] Qin G Mao H Zheng J Sia S Liu Y Chan P. Phosphoantigen-expanded human γδ T cells display potent cytotoxicity against monocyte-derived macrophages infected with human and avian influenza viruses. J Infect Dis (2009) 200:858–65. doi: 10.1086/605413 PMC7110194 19656068

[29] Dieli F Troye-Blomberg M Ivanyi J Fournie J Krensky A Bonneville M. Granulysin-dependent killing of intracellular and extracellular mycobacterium tuberculosis by Vγ9/Vδ2 T lymphocytes. J Infect Dis (2001) 184:1082–5. doi: 10.1086/323600 11574927

[30] Toulon A Breton L Taylor K Tenenhaus M Bhavsar D Lanigan C. A role for human skin–resident T cells in wound healing. J Exp Med (2009) 206:743–50. doi: 10.1084/jem.20081787 PMC2715110 19307328

[31] Kohlgruber A Gal-Oz S LaMarche N Shimazaki M Duquette D Koay H. γδ T cells producing interleukin-17A regulate adipose regulatory T cell homeostasis and thermogenesis. Nat Immunol (2018) 19:464–74. doi: 10.1038/s41590-018-0094-2 PMC8299914 29670241

[32] Darrasse-Jèze G Deroubaix S Mouquet H Victora GD Eisenreich T Yao KH. Feedback control of regulatory T cell homeostasis by dendritic cells *in vivo* . J Exp Med (2009) 206:1853–62. doi: 10.1084/JEM.20090746 PMC2737156 19667061

[33] Patente TA Pinho MP Oliveira AA Evangelista GCM Bergami-Santos PC Barbuto JAM. Human dendritic cells: Their heterogeneity and clinical application potential in cancer immunotherapy. Front Immunol (2019) 10:3176. doi: 10.3389/fimmu.2018.03176 PMC6348254 30719026

[34] Embgenbroich M Burgdorf S. Current concepts of antigen cross-presentation. Front Immunol (2018) 9:1643. doi: 10.3389/fimmu.2018.01643 30061897 PMC6054923

[35] Jang MH Sougawa N Tanaka T Hirata T Hiroi T Tohya K. CCR7 is critically important for migration of dendritic cells in intestinal lamina propria to mesenteric lymph nodes. J Immunol (2006) 176:803–10. doi: 10.4049/jimmunol.176.2.803 16393963

[36] Förster R Schubel A Breitfeld D Kremmer E Renner-Müller I Wolf E. CCR7 coordinates the primary immune response by establishing functional microenvironments in secondary lymphoid organs. Cell (1999) 99:23–33. doi: 10.1016/S0092-8674(00)80059-8 10520991

[37] Stagg AJ. Intestinal dendritic cells in health and gut inflammation. Front Immunol (2018) 9:2883. doi: 10.3389/fimmu.2018.02883 30574151 PMC6291504

[38] Zenclussen AC Hämmerling GJ. Cellular regulation of the uterine microenvironment that enables embryo implantation. Front Immunol (2015) 6:321. doi: 10.3389/fimmu.2015.00321 26136750 PMC4470084

[39] Chang SY Ko HJ Kweon MN. Mucosal dendritic cells shape mucosal immunity. Exp Mol Med (2014) 46:1–7. doi: 10.1038/emm.2014.16 PMC3972789 24626170

[40] Hemmi H Akira S. TLR signalling and the function of dendritic cells. Chem Immunol Allergy (2005) 86:120–35. doi: 10.1159/000086657 15976491

[41] Hubo M Trinschek B Kryczanowsky F Tüttenberg A Steinbrink K Jonuleit H. Costimulatory molecules on immunogenic versus tolerogenic human dendritic cells. Front Immunol (2013) 4:82. doi: 10.3389/fimmu.2013.00082 23565116 PMC3615188

[42] Guilliams M Ginhoux F Jakubzick C Naik S Onai N Schraml BU. Dendritic cells, monocytes and macrophages: A unified nomenclature based on ontogeny. Nat Rev Immunol (2014) 14:571–8. doi: 10.1038/nri3712 PMC4638219 25033907

[43] Doebel T Voisin B Nagao K. Langerhans cells – the macrophage in dendritic cell clothing. Trends Immunol (2017) 38:817–28. doi: 10.1016/j.it.2017.06.008 28720426

[44] Villani AC Satija R Reynolds G Sarkizova S Shekhar K Fletcher J. Single-cell RNA-seq reveals new types of human blood dendritic cells, monocytes, and progenitors. Science (2017) 356:eaah4573. doi: 10.1126/science.aah4573 28428369 PMC5775029

[45] Villar J Segura E. Decoding the heterogeneity of human dendritic cell subsets. Trends Immunol (2020) 41:1062–71. doi: 10.1016/j.it.2020.10.002 33250080

[46] Valladeau J Ravel O Dezutter-Dambuyant C Moore K Kleijmeer M Liu Y. Langerin, a novel c-type lectin specific to langerhans cells, is an endocytic receptor that induces the formation of birbeck granules. Immunity (2000) 12:71–81. doi: 10.1016/S1074-7613(00)80160-0 10661407

[47] Posch W Lass-Flörl C Wilflingseder D. Generation of human monocyte-derived dendritic cells from whole blood. J Vis Exp (2016) 118:54968. doi: 10.3791/54968 PMC5226452 28060313

[48] Varol C Landsman L Fogg DK Greenshtein L Gildor B Margalit R. Monocytes give rise to mucosal, but not splenic, conventional dendritic cells. J Exp Med (2007) 204:171–80. doi: 10.1084/jem.20061011 PMC2118434 17190836

[49] León B López-Bravo M Ardavín C. Monocyte-derived dendritic cells formed at the infection site control the induction of protective T helper 1 responses against leishmania. Immunity (2007) 26:519–31. doi: 10.1016/j.immuni.2007.01.017 17412618

[50] Segura E Touzot M Bohineust A Cappuccio A Chiocchia G Hosmalin A. Human inflammatory dendritic cells induce Th17 cell differentiation. Immunity (2013) 38:336–48. doi: 10.1016/J.IMMUNI.2012.10.018 23352235

[51] Coillard A Segura E. *In vivo* differentiation of human monocytes. Front Immunol (2019) 10:1907. doi: 10.3389/fimmu.2019.01907 31456804 PMC6700358

[52] Mair F Liechti T. Comprehensive phenotyping of human dendritic cells and monocytes. Cytometry Part A (2021) 99:231–42. doi: 10.1002/cyto.a.24269 33200508

[53] Dutertre C-A Becht E Irac SE Khalilnezhad A Narang V Khalilnezhad S. Single-cell analysis of human mononuclear phagocytes reveals subset-defining markers and identifies circulating inflammatory dendritic cells. Immunity (2019) 51:573–589.e8. doi: 10.1016/j.immuni.2019.08.008 31474513

[54] James KR Gomes T Elmentaite R Kumar N Gulliver EL King HW. Distinct microbial and immune niches of the human colon. Nat Immunol (2020) 21:343–53. doi: 10.1038/s41590-020-0602-z PMC7212050 32066951

[55] Trifonova RT Lieberman J van Baarle D. Distribution of immune cells in the human cervix and implications for HIV transmission. Am J Reprod Immunol (2014) 71:252–64. doi: 10.1111/aji.12198 PMC3943534 24410939

[56] Xue D Tabib T Morse C Lafyatis R. Transcriptome landscape of myeloid cells in human skin reveals diversity, rare populations and putative DC progenitors. J Dermatol Sci (2020) 97:41–9. doi: 10.1016/j.jdermsci.2019.11.012 31836271

[57] Cytlak U Resteu A Pagan S Green K Milne P Maisuria S. Differential IRF8 transcription factor requirement defines two pathways of dendritic cell development in humans. Immunity (2020) 53:353–370.e8. doi: 10.1016/j.immuni.2020.07.003 32735845 PMC7447982

[58] Guilliams M Dutertre C-A Scott CL McGovern N Sichien D Chakarov S. Unsupervised high-dimensional analysis aligns dendritic cells across tissues and species. Immunity (2016) 45:669–84. doi: 10.1016/j.immuni.2016.08.015 PMC5040826 27637149

[59] Fedorenko A Lishko PV Kirichok Y. Mechanism of fatty-Acid-Dependent UCP1 uncoupling in brown fat mitochondria. Cell (2012) 151:400–13. doi: 10.1016/J.CELL.2012.09.010 PMC3782081 23063128

[60] Wu J Boström P Sparks LM Ye L Choi JH Giang AH. Beige adipocytes are a distinct type of thermogenic fat cell in mouse and human. Cell (2012) 150:366–76. doi: 10.1016/J.CELL.2012.05.016 PMC3402601 22796012

[61] Bapat SP Liang Y Zheng Y. Characterization of immune cells from adipose tissue. Curr Protoc Immunol (2019) 126:e86. doi: 10.1002/CPIM.86 31483101 PMC6814145

[62] Lee MW Odegaard JI Mukundan L Qiu Y Molofsky AB Nussbaum JC. Activated type 2 innate lymphoid cells regulate beige fat biogenesis. Cell (2015) 160:74–87. doi: 10.1016/J.CELL.2014.12.011 25543153 PMC4297518

[63] Brestoff JR Kim BS Saenz SA Stine RR Monticelli LA Sonnenberg GF. Group 2 innate lymphoid cells promote beiging of white adipose tissue and limit obesity. Nature (2014) 519:242–6. doi: 10.1038/nature14115 PMC4447235 25533952

[64] Kang K Reilly SM Karabacak V Gangl MR Fitzgerald K Hatano B. Adipocyte-derived Th2 cytokines and myeloid PPARδ regulate macrophage polarization and insulin sensitivity. Cell Metab (2008) 7:485–95. doi: 10.1016/J.CMET.2008.04.002 PMC2586840 18522830

[65] Nishimura S Manabe I Nagasaki M Eto K Yamashita H Ohsugi M. CD8+ effector T cells contribute to macrophage recruitment and adipose tissue inflammation in obesity. Nat Med (2009) 15:914–20. doi: 10.1038/nm.1964 19633658

[66] Feuerer M Herrero L Cipolletta D Naaz A Wong J Nayer A. Lean, but not obese, fat is enriched for a unique population of regulatory T cells that affect metabolic parameters. Nat Med (2009) 15:930–9. doi: 10.1038/nm.2002 PMC3115752 19633656

[67] Liu Z Hu X Liang Y Yu J Li H Shokhirev MN. Glucocorticoid signaling and regulatory T cells cooperate to maintain the hair-follicle stem-cell niche. Nat Immunol (2022) 23:1086–97. doi: 10.1038/s41590-022-01244-9 PMC9283297 35739197

[68] Collins N Jiang X Zaid A Macleod BL Li J Park CO. Skin CD4+ memory T cells exhibit combined cluster-mediated retention and equilibration with the circulation. Nat Commun (2016) 7:11514. doi: 10.1038/ncomms11514 27160938 PMC4866325

[69] Adachi T Kobayashi T Sugihara E Yamada T Ikuta K Pittaluga S. Hair follicle–derived IL-7 and IL-15 mediate skin-resident memory T cell homeostasis and lymphoma. Nat Med (2015) 21:1272–9. doi: 10.1038/nm.3962 PMC4636445 26479922

[70] Rodriguez RS Pauli ML Neuhaus IM Yu SS Arron ST Harris HW. Memory regulatory T cells reside in human skin. J Clin Invest (2014) 124:1027–36. doi: 10.1172/JCI72932 PMC3934172 24509084

[71] Tordesillas L Lozano-Ojalvo D Dunkin D Mondoulet L Agudo J Merad M. PDL2+ CD11b+ dermal dendritic cells capture topical antigen through hair follicles to prime LAP+ tregs. Nat Commun (2018) 9:5238. doi: 10.1038/s41467-018-07716-7 30531969 PMC6286332

[72] Zhu J Koelle DM Cao J Vazquez J Meei LH Hladik F. Virus-specific CD8+ T cells accumulate near sensory nerve endings in genital skin during subclinical HSV-2 reactivation. J Exp Med (2007) 204:595–603. doi: 10.1084/jem.20061792 17325200 PMC2137910

[73] Kolter J Feuerstein R Zeis P Hagemeyer N Paterson N d’Errico P. A subset of skin macrophages contributes to the surveillance and regeneration of local nerves. Immunity (2019) 50:1482–1497.e7. doi: 10.1016/j.immuni.2019.05.009 31201094

[74] Nagao K Ginhoux F Leitner WW Motegi S-I Bennett CL Clausen BE. Murine epidermal langerhans cells and langerin-expressing dermal dendritic cells are unrelated and exhibit distinct functions. Proc Natl Acad Sci U.S.A. (2009) 106:3312–7. doi: 10.1073/pnas.0807126106 PMC2651335 19218433

[75] Vishwanath M Nishibu A Saeland S Ward BR Mizumoto N Ploegh HL. Development of intravital intermittent confocal imaging system for studying langerhans cell turnover. J Invest Dermatol (2006) 126:2452–7. doi: 10.1038/sj.jid.5700448 16794586

[76] Figdor CG van Kooyk Y Adema GJ. C-type lectin receptors on dendritic cells and langerhans cells. Nat Rev Immunol (2002) 2:77–84. doi: 10.1038/nri723 11910898

[77] Stössel H Koch F Kämpgen E Stöger P Lenz A Heufler C. Disappearance of certain acidic organelles (endosomes and langerhans cell granules) accompanies loss of antigen processing capacity upon culture of epidermal langerhans cells. J Exp Med (1990) 172:1471–82. doi: 10.1084/jem.172.5.1471 PMC2188657 2230653

[78] Fujita H Nograles KE Kikuchi T Gonzalez J Carucci JA Krueger JG. Human langerhans cells induce distinct IL-22-producing CD4+ T cells lacking IL-17 production. Proc Natl Acad Sci U.S.A. (2009) 106:21795–800. doi: 10.1073/pnas.0911472106 PMC2799849 19996179

[79] Péguet-Navarro J Furio L Briotet I Journeaux A Billard H. Human langerhans cells are more efficient than CD14–CD1c+ dermal dendritic cells at priming naive CD4+ T cells. J Invest Dermatol (2010) 130:1345–54. doi: 10.1038/JID.2009.424 20107482

[80] Klechevsky E Morita R Liu M Cao Y Coquery S Thompson-Snipes LA. Functional specializations of human epidermal langerhans cells and CD14+ dermal dendritic cells. Immunity (2008) 29:497–510. doi: 10.1016/J.IMMUNI.2008.07.013 18789730 PMC2688399

[81] Seneschal J Clark RA Gehad A Baecher-Allan CM Kupper TS. Human epidermal langerhans cells maintain immune homeostasis in skin by activating skin resident regulatory T cells. Immunity (2012) 36:873–84. doi: 10.1016/J.IMMUNI.2012.03.018 PMC3716276 22560445

[82] Ouchi T Kubo A Yokouchi M Adachi T Kobayashi T Kitashima DY. Langerhans cell antigen capture through tight junctions confers preemptive immunity in experimental staphylococcal scalded skin syndrome. J Exp Med (2011) 208:2607–13. doi: 10.1084/jem.20111718 PMC3244045 22143886

[83] Gosselin D Link VM Romanoski CE Fonseca GJ Eichenfield DZ Spann NJ. Environment drives selection and function of enhancers controlling tissue-specific macrophage identities. Cell (2014) 159:1327–40. doi: 10.1016/J.CELL.2014.11.023 PMC4364385 25480297

[84] Lucas T Waisman A Ranjan R Roes J Krieg T Müller W. Differential roles of macrophages in diverse phases of skin repair. J Immunol (2010) 184:3964–77. doi: 10.4049/JIMMUNOL.0903356 20176743

[85] Ishida Y Gao J-L Murphy PM. Chemokine receptor CX3CR1 mediates skin wound healing by promoting macrophage and fibroblast accumulation and function. J Immunol (2008) 180:569–79. doi: 10.4049/JIMMUNOL.180.1.569 18097059

[86] Zaba LC Krueger JG Lowes MA. Resident and “Inflammatory” dendritic cells in human skin. J Invest Dermatol (2009) 129:302–8. doi: 10.1038/JID.2008.225 PMC2746703 18685620

[87] Zaba LC Fuentes-Duculan J Steinman RM Krueger JG Lowes MA. Normal human dermis contains distinct populations of CD11c+BDCA-1+ dendritic cells and CD163+FXIIIA+ macrophages. J Clin Invest (2007) 117:2517–25. doi: 10.1172/JCI32282 PMC1957542 17786242

[88] Haniffa M Gunawan M Jardine L. Human skin dendritic cells in health and disease. J Dermatol Sci (2015) 77:85–92. doi: 10.1016/j.jdermsci.2014.08.012 25301671 PMC4728191

[89] Haniffa M Ginhoux F Wang X-N Bigley V Abel M Dimmick I. Differential rates of replacement of human dermal dendritic cells and macrophages during hematopoietic stem cell transplantation. J Exp Med (2009) 206:371–85. doi: 10.1084/jem.20081633 PMC2646566 19171766

[90] Britschgi MR Favre S Luther SA. CCL21 is sufficient to mediate DC migration, maturation and function in the absence of CCL19. Eur J Immunol (2010) 40:1266–71. doi: 10.1002/eji.200939921 20201039

[91] Link A Vogt TK Favre S Britschgi MR Acha-Orbea H Hinz B. Fibroblastic reticular cells in lymph nodes regulate the homeostasis of naive T cells. Nat Immunol (2007) 8:1255–65. doi: 10.1038/ni1513 17893676

[92] Nanno M Shiohara T Yamamoto H Kawakami K Ishikawa H. γδ T cells: firefighters or fire boosters in the front lines of inflammatory responses. Immunol Rev (2007) 215:103–13. doi: 10.1111/J.1600-065X.2006.00474.X 17291282

[93] Rei W Ahmed G Chao Y SL L TJ E Christoph S. Human skin is protected by four functionally and phenotypically discrete populations of resident and recirculating memory T cells. Sci Transl Med (2015) 7:279ra39–279ra39. doi: 10.1126/scitranslmed.3010302 PMC4425193 25787765

[94] Allison TJ Winter CC Fournié JJ Bonneville M Garboczi DN. Structure of a human γδ T-cell antigen receptor. Nature (2001) 411:820–4. doi: 10.1038/35081115 11459064

[95] Bukowski JF Morita CT Brenner MB. Human γδ T cells recognize alkylamines derived from microbes, edible plants, and tea: Implications for innate immunity. Immunity (1999) 11:57–65. doi: 10.1016/S1074-7613(00)80081-3 10435579

[96] Bürk MR Mori L de Libero G. Human Vγ9-Vδ2 cells are stimulated in a crossreactive fashion by a variety of phosphorylated metabolites. Eur J Immunol (1995) 25:2052–8. doi: 10.1002/EJI.1830250737 7621879

[97] Fichtner AS Ravens S Prinz I. Human γδ TCR repertoires in health and disease. Cells (2020) 9:800. doi: 10.3390/CELLS9040800 PMC7226320 32225004

[98] Sharp LL Jameson JM Cauvi G Havran WL. Dendritic epidermal T cells regulate skin homeostasis through local production of insulin-like growth factor 1. Nat Immunol (2004) 6:73–9. doi: 10.1038/ni1152 15592472

[99] Pan Y Tian T Park CO Lofftus SY Mei S Liu X. Survival of tissue-resident memory T cells requires exogenous lipid uptake and metabolism. Nature (2017) 543:252–6. doi: 10.1038/NATURE21379 PMC5509051 28219080

[100] Mohammed J Beura LK Bobr A Astry B Chicoine B Kashem SW. Stromal cells control the epithelial residence of DCs and memory T cells by regulated activation of TGF-β. Nat Immunol (2016) 17:414–21. doi: 10.1038/ni.3396 PMC5135085 26901152

[101] CR A Rei W E Christoph S TM C Natalie A DA A. Skin effector memory T cells do not recirculate and provide immune protection in alemtuzumab-treated CTCL patients. Sci Transl Med (2012) 4:117ra7–7. doi: 10.1126/scitranslmed.3003008 PMC3373186 22261031

[102] Santamaria Babi LF Moser R Perez Soler MT Picker LJ Blaser K Hauser C. Migration of skin-homing T cells across cytokine-activated human endothelial cell layers involves interaction of the cutaneous lymphocyte-associated antigen (CLA), the very late antigen-4 (VLA-4), and the lymphocyte function-associated antigen-1 (LFA-1). J Immunol (1995) 154:1543–50. 7836740

[103] Mitoma J Bao X Petryanik B Schaerli P Gauguet JM Yu SY. Critical functions of n-glycans in l-selectin-mediated lymphocyte homing and recruitment. Nat Immunol (2007) 8:409–18. doi: 10.1038/ni1442 17334369

[104] Rossiter H van Reijsen F Mudde GC Kalthoff F Bruijnzeel-Koomen CA Picker LJ. Skin disease-related T cells bind to endothelial selectins: Expression of cutaneous lymphocyte antigen (CLA) predicts e-selectin but not p-selectin binding. Eur J Immunol (1994) 24:205–10. doi: 10.1002/EJI.1830240132 7517361

[105] Fuhlbrigge RC David Kieffer J Armerding D Kupper TS. Cutaneous lymphocyte antigen is a specialized form of PSGL-1 expressed on skin-homing T cells. Nature (1997) 389:978–81. doi: 10.1038/40166 9353122

[106] Campbell JJ Murphy KE Kunkel EJ Brightling CE Soler D Shen Z. CCR7 expression and memory T cell diversity in humans. J Immunol (2001) 166:877. doi: 10.4049/jimmunol.166.2.877 11145663

[107] Baekkevold ES Yamanaka T Palframan RT Carlsen HS Reinholt FP von Andrian UH. The Ccr7 ligand ELC (Ccl19) is transcytosed in high endothelial venules and mediates T cell recruitment. J Exp Med (2001) 193:1105–12. doi: 10.1084/jem.193.9.1105 PMC2193428 11342595

[108] Campbell JJ Haraldsen G Pan J Rottman J Qin S Ponath P. The chemokine receptor CCR4 in vascular recognition by cutaneous but not intestinal memory T cells. Nature (1999) 400:776–80. doi: 10.1038/23495 10466728

[109] Campbell DJ Butcher EC. Rapid acquisition of tissue-specific homing phenotypes by CD4+ T cells activated in cutaneous or mucosal lymphoid tissues. J Exp Med (2002) 195:135–41. doi: 10.1084/jem.20011502 PMC2196018 11781372

[110] Sallusto F Geginat J Lanzavecchia A. Central memory and effector memory T cell subsets: Function, generation, and maintenance. Annu Rev Immunol (2004) 22:745–63. doi: 10.1146/annurev.immunol.22.012703.104702 15032595

[111] Park CO Kupper TS. The emerging role of resident memory T cells in protective immunity and inflammatory disease. Nat Med (2015) 21:688–97. doi: 10.1038/nm.3883 PMC4640452 26121195

[112] Zaid A Hor JL Christo SN Groom JR Heath WR Mackay LK. Chemokine receptor–dependent control of skin tissue–resident memory T cell formation. J Immunol (2017) 199:2451–9. doi: 10.4049/jimmunol.1700571 28855310

[113] Zaid A Mackay LK Rahimpour A Braun A Veldhoen M Carbone FR. Persistence of skin-resident memory T cells within an epidermal niche. Proc Natl Acad Sci U.S.A. (2014) 111:5307–12. doi: 10.1073/PNAS.1322292111 PMC3986170 24706879

[114] Beura LK Mitchell JS Thompson EA Schenkel JM Mohammed J Wijeyesinghe S. Intravital mucosal imaging of CD8+ resident memory T cells shows tissue-autonomous recall responses that amplify secondary memory. Nat Immunol (2018) 19:173–82. doi: 10.1038/s41590-017-0029-3 PMC5896323 29311694

[115] Egawa G Honda T Tanizaki H Doi H Miyachi Y Kabashima K. *In vivo* imaging of t-cell motility in the elicitation phase of contact hypersensitivity using two-photon microscopy. J Invest Dermatol (2011) 131:977–9. doi: 10.1038/jid.2010.386 21248770

[116] Honda T Egen JG Lämmermann T Kastenmüller W Torabi-Parizi P Germain RN. Tuning of antigen sensitivity by T cell receptor-dependent negative feedback controls T cell effector function in inflamed tissues. Immunity (2014) 40:235–47. doi: 10.1016/j.immuni.2013.11.017 PMC4792276 24440150

[117] Ariotti S Beltman JB Chodaczek G Hoekstra ME van Beek AE Gomez-Eerland R. Tissue-resident memory CD8+ T cells continuously patrol skin epithelia to quickly recognize local antigen. Proc Natl Acad Sci U.S.A. (2012) 109:19739–44. doi: 10.1073/PNAS.1208927109 PMC3511734 23150545

[118] Dijkgraaf FE Matos TR Hoogenboezem M Toebes M Vredevoogd DW Mertz M. Tissue patrol by resident memory CD8+ T cells in human skin. Nat Immunol (2019) 20:756–64. doi: 10.1038/s41590-019-0404-3 31110315

[119] Gebhardt T Whitney PG Zaid A MacKay LK Brooks AG Heath WR. Different patterns of peripheral migration by memory CD4+ and CD8+ T cells. Nature (2011) 477:216–9. doi: 10.1038/nature10339 21841802

[120] Gebhardt T Mueller SN Heath WR Carbone FR. Peripheral tissue surveillance and residency by memory T cells. Trends Immunol (2013) 34:27–32. doi: 10.1016/J.IT.2012.08.008 23036434

[121] Flacher V Tripp CH Haid B Kissenpfennig A Malissen B Stoitzner P. Skin langerin+ dendritic cells transport intradermally injected anti–DEC-205 antibodies but are not essential for subsequent cytotoxic CD8+ T cell responses. J Immunol (2012) 188:2146–55. doi: 10.4049/JIMMUNOL.1004120 PMC3288813 22291181

[122] Tripp CH Sparber F Hermans IF Romani N Stoitzner P. Glycolipids injected into the skin are presented to NKT cells in the draining lymph node independently of migratory skin dendritic cells. J Immunol (2009) 182:7644–54. doi: 10.4049/JIMMUNOL.0900134 19494288

[123] Park SL Zaid A Hor JL Christo SN Prier JE Davies B. Local proliferation maintains a stable pool of tissue-resident memory T cells after antiviral recall responses. Nat Immunol (2018) 19:183–91. doi: 10.1038/s41590-017-0027-5 29311695

[124] Haniffa M Shin A Bigley V McGovern N Teo P See P. Human tissues contain CD141 hi cross-presenting dendritic cells with functional homology to mouse CD103 + nonlymphoid dendritic cells. Immunity (2012) 37:60–73. doi: 10.1016/j.immuni.2012.04.012 22795876 PMC3476529

[125] den Haan JMM Bevan MJ. Constitutive versus activation-dependent cross-presentation of immune complexes by CD8(+) and CD8(-) dendritic cells *in vivo* . J Exp Med (2002) 196:817–27. doi: 10.1084/JEM.20020295 PMC2194052 12235214

[126] Miragaia RJ Gomes T Chomka A Jardine L Riedel A Hegazy AN. Single-cell transcriptomics of regulatory T cells reveals trajectories of tissue adaptation. Immunity (2019) 50:493–504.e7. doi: 10.1016/j.immuni.2019.01.001 30737144 PMC6382439

[127] Clark RA Kupper TS. IL-15 and dermal fibroblasts induce proliferation of natural regulatory T cells isolated from human skin. Blood (2007) 109:194–202. doi: 10.1182/BLOOD-2006-02-002873 16968902 PMC1785078

[128] Clark RA Huang SJ Murphy GF Mollet IG Hijnen D Muthukuru M. Human squamous cell carcinomas evade the immune response by down-regulation of vascular e-selectin and recruitment of regulatory T cells. J Exp Med (2008) 205:2221–34. doi: 10.1084/JEM.20071190 PMC2556796 18794336

[129] Hirahara K Liu L Clark RA Yamanaka K Fuhlbrigge RC Kupper TS. The majority of human peripheral blood CD4+CD25highFoxp3+ regulatory T cells bear functional skin-homing receptors. J Immunol (2006) 177:4488–94. doi: 10.4049/JIMMUNOL.177.7.4488 16982885

[130] Rosenblum MD Gratz IK Paw JS Lee K Marshak-Rothstein A Abbas AK. Response to self-antigen imprints regulatory memory in tissues. Nature (2011) 480:538–42. doi: 10.1038/nature10664 PMC3263357 22121024

[131] Arpaia N Green JA Moltedo B Arvey A Hemmers S Yuan S. A distinct function of regulatory T cells in tissue protection. Cell (2015) 162:1078–89. doi: 10.1016/j.cell.2015.08.021 PMC4603556 26317471

[132] Nosbaum A Prevel N Truong H-A Mehta P Ettinger M Scharschmidt TC. Cutting edge: Regulatory T cells facilitate cutaneous wound healing. J Immunol (2016) 196:2010–4. doi: 10.4049/JIMMUNOL.1502139 PMC4761457 26826250

[133] Ali N Zirak B Rodriguez RS Pauli ML Truong HA Lai K. Regulatory T cells in skin facilitate epithelial stem cell differentiation. Cell (2017) 169:1119–1129.e11. doi: 10.1016/J.CELL.2017.05.002 28552347 PMC5504703

[134] Holtmeier W Kabelitz D. γδ T cells link innate and adaptive immune responses. Chem Immunol Allergy (2005) 86:151–83. doi: 10.1159/000086659 15976493

[135] Xiong N Raulet DH. Development and selection of γδ T cells. Immunol Rev (2007) 215:15–31. doi: 10.1111/J.1600-065X.2006.00478.X 17291276

[136] Havran WL Allison JP. Developmentally ordered appearance of thymocytes expressing different T-cell antigen receptors. Nature (1988) 335:443–5. doi: 10.1038/335443a0 2458531

[137] Spada FM Grant EP Peters PJ Sugita M Melián A Leslie DS. Self-recognition of CD1 by gamma/delta T cells: implications for innate immunity. J Exp Med (2000) 191:937–48. doi: 10.1084/JEM.191.6.937 PMC2193122 10727456

[138] Paradis TJ Cole SH Nelson RT Gladue RP. Essential role of CCR6 in directing activated T cells to the skin during contact hypersensitivity. J Invest Dermatol (2008) 128:628–33. doi: 10.1038/SJ.JID.5701055 17882271

[139] Schmuth M Neyer S Rainer C Grassegger A Fritsch P Romani N. Expression of the c-c chemokine MIP-3α/CCL20 in human epidermis with impaired permeability barrier function. Exp Dermatol (2002) 11:135–42. doi: 10.1034/J.1600-0625.2002.110205.X 11994140

[140] Sudo T Nishikawa S Ohno N Akiyama N Tamakoshi M Yoshida H. Expression and function of the interleukin 7 receptor in murine lymphocytes. Proc Natl Acad Sci U.S.A. (1993) 90:9125–9. doi: 10.1073/PNAS.90.19.9125 PMC47514 8415665

[141] Cao X Shores EW Hu-LI J Ft Anver M Kelsall BL SM R. Defective lymphoid development in mice lacking expression of the common cytokine receptor y chain. Immunity (1995) 2:223–36. doi: 10.1016/1074-7613(95)90047-0 7697543

[142] Lodolce JP Boone DL Chai S Swain RE Dassopoulos T Trettin S. IL-15 receptor maintains lymphoid homeostasis by supporting lymphocyte homing and proliferation. Immunity (1998) 9:669–76. doi: 10.1016/S1074-7613(00)80664-0 9846488

[143] Schlickum S Sennefelder H Friedrich M Harms G Lohse MJ Kilshaw P. Integrin αE(CD103)β7 influences cellular shape and motility in a ligand-dependent fashion. Blood (2008) 112:619–25. doi: 10.1182/BLOOD-2008-01-134833 18492951

[144] Ribot JC DeBarros A Silva-Santos B. Searching for “signal 2”: Costimulation requirements of γδ T cells. Cell Mol Life Sci (2011) 68:2345–55. doi: 10.1007/S00018-011-0698-2 PMC11115137 21541698

[145] Das H Sugita M Brenner MB. Mechanisms of Vδ1 γδ T cell activation by microbial components. J Immunol (2004) 172:6578–86. doi: 10.4049/JIMMUNOL.172.11.6578 15153472

[146] McAlister MSB Mott HR van der Merwe PA Campbell ID Davis SJ Driscoll PC. NMR analysis of interacting soluble forms of the cell–cell recognition molecules CD2 and CD48†. Biochemistry (1996) 35:5982–91. doi: 10.1021/BI952756U 8634239

[147] Roy J Audette M Tremblay MJ. Intercellular adhesion molecule-1 (ICAM-1) gene expression in human T cells is regulated by phosphotyrosyl phosphatase activity. involvement of NF-kappaB, ets, and palindromic interferon-gamma-responsive element-binding sites. J Biol Chem (2001) 276:14553–61. doi: 10.1074/JBC.M005067200 11278281

[148] Jameson J Ugarte K Chen N Yachi P Fuchs E Boismenu R. A role for skin γδ T cells in wound repair. Science (2002) 296:747–9. doi: 10.1126/SCIENCE.1069639 11976459

[149] Bauer S Groh V Wu J Steinle A Phillips JH Lanier LL. Activation of NK cells and T cells by NKG2D, a receptor for stress-inducible MICA. Science (1999) 285:727–9. doi: 10.1126/SCIENCE.285.5428.727 10426993

[150] Ebert LM Meuter S Moser B. Homing and function of human skin γδ T cells and NK cells: Relevance for tumor surveillance. J Immunol (2006) 176:4331–6. doi: 10.4049/JIMMUNOL.176.7.4331 16547270

[151] Chow Z Mueller SN Deane JA Hickey MJ. Dermal regulatory T cells display distinct migratory behavior that is modulated during adaptive and innate inflammation. J Immunol (2013) 191:3049–56. doi: 10.4049/JIMMUNOL.1203205 23940277

[152] Dubois Declercq S Pouliot R. Promising new treatments for psoriasis. Sci World J (2013) 2013:980419. doi: 10.1155/2013/980419 PMC3713318 23935446

[153] Hawkes JE Chan TC Krueger JG. Psoriasis pathogenesis and the development of novel targeted immune therapies. J Allergy Clin Immunol (2017) 140:645–53. doi: 10.1016/J.JACI.2017.07.004 PMC5600287 28887948

[154] Lee E Trepicchio WL Oestreicher JL Pittman D Wang F Chamian F. Increased expression of interleukin 23 p19 and p40 in lesional skin of patients with psoriasis vulgaris. J Exp Med (2004) 199:125–30. doi: 10.1084/JEM.20030451 PMC1887731 14707118

[155] Onishi RM Gaffen SL. Interleukin-17 and its target genes: Mechanisms of interleukin-17 function in disease. Immunology (2010) 129:311–21. doi: 10.1111/J.1365-2567.2009.03240.X PMC2826676 20409152

[156] Bosè F Petti L Diani M Moscheni C Molteni S Altomare A. Inhibition of CCR7/CCL19 axis in lesional skin is a critical event for clinical remission induced by TNF blockade in patients with psoriasis. Am J Pathol (2013) 183:413–21. doi: 10.1016/j.ajpath.2013.04.021 23731727

[157] Cai Y Shen X Ding C Qi C Li K Li X. Pivotal role of dermal IL-17-Producing γδ T cells in skin inflammation. mmunity (2011) 35:596–610. doi: 10.1016/J.IMMUNI.2011.08.001 PMC3205267 21982596

[158] Laggner U Meglio P Perera GK Hundhausen C Lacy KE Ali N. Identification of a novel proinflammatory human skin-homing Vγ9Vδ2 T cell subset with a potential role in psoriasis. J Immunol (2011) 187:2783–93. doi: 10.4049/JIMMUNOL.1100804 PMC3187621 21813772

[159] Mavropoulos A Rigopoulou EI Liaskos C Bogdanos DP Sakkas LI. The role of p38 mapk in the aetiopathogenesis of psoriasis and psoriatic arthritis. Clin Dev Immunol (2013) 2013:569751. doi: 10.1155/2013/569751 24151518 PMC3787653

[160] Zheng T Zhao W Li H Xiao S Hu R Han M. P38α signaling in langerhans cells promotes the development of IL-17-producing T cells and psoriasiform skin inflammation. Sci Signal (2018) 11:1685. doi: 10.1126/SCISIGNAL.AAO1685 29535261

[161] Wolk K Witte E Wallace E Döcke WD Kunz S Asadullah K. IL-22 regulates the expression of genes responsible for antimicrobial defense, cellular differentiation, and mobility in keratinocytes: A potential role in psoriasis. Eur J Immunol (2006) 36:1309–23. doi: 10.1002/EJI.200535503 16619290

[162] Novak N Bieber T Leung DYM. Immune mechanisms leading to atopic dermatitis. J Allergy Clin Immunol (2003) 112:S128–39. doi: 10.1016/j.jaci.2003.09.032 14657843

[163] Brown SJ Irwin McLean WH. Eczema genetics: Current state of knowledge and future goals. J Invest Dermatol (2009) 129:543–52. doi: 10.1038/jid.2008.413 19209157

[164] Clausen ML Edslev SM Andersen PS Clemmensen K Krogfelt KA Agner T. Staphylococcus aureus colonization in atopic eczema and its association with filaggrin gene mutations. Br J Dermatol (2017) 177:1394–400. doi: 10.1111/BJD.15470 28317091

[165] Kong HH Oh J Deming C Conlan S Grice EA Beatson MA. Temporal shifts in the skin microbiome associated with disease flares and treatment in children with atopic dermatitis. Genome Res (2012) 22:850–9. doi: 10.1101/GR.131029.111 PMC3337431 22310478

[166] Piet B de Bree GJ Smids-Dierdorp BS van der Loos CM Remmerswaal EBM von der Thüsen JH. CD8+ T cells with an intraepithelial phenotype upregulate cytotoxic function upon influenza infection in human lung. J Clin Invest (2011) 121:2254–63. doi: 10.1172/JCI44675 PMC3104744 21537083

[167] Purwar R Campbell J Murphy G Richards WG Clark RA Kupper TS. Resident memory T cells (TRM) are abundant in human lung: Diversity, function, and antigen specificity. PloS One (2011) 6:e16245. doi: 10.1371/JOURNAL.PONE.0016245 21298112 PMC3027667

[168] Jozwik A Habibi MS Paras A Zhu J Guvenel A Dhariwal J. RSV-Specific airway resident memory CD8+ T cells and differential disease severity after experimental human infection. Nat Commun (2015) 6:10224. doi: 10.1038/ncomms10224 26687547 PMC4703893

[169] Verjans GMGM Hintzen RQ van Dun JM Poot A Milikan JC Laman JD. Selective retention of herpes simplex virus-specific T cells in latently infected human trigeminal ganglia. Proc Natl Acad Sci U.S.A. (2007) 104:3496–501. doi: 10.1073/pnas.0610847104 PMC1805572 17360672

[170] Zhu J Peng T Johnston C Phasouk K Kask AS Klock A. Immune surveillance by CD8αα+ skin-resident t cells in human herpes virus infection. Nature (2013) 497:494–7. doi: 10.1038/nature12110 PMC3663925 23657257

[171] Hislop AD Kuo M Drake-Lee AB Akbar AN Bergler W Hammerschmitt N. Tonsillar homing of Epstein-Barr virus–specific CD8+ T cells and the virus-host balance. J Clin Invest (2005) 115:2546–55. doi: 10.1172/JCI24810 PMC1187932 16110323

[172] Woodberry T Suscovich TJ Henry LM August M Waring MT Kaur A. αEβ7 (CD103) expression identifies a highly active, tonsil-resident effector-memory CTL population. J Immunol (2005) 175:4355–62. doi: 10.4049/JIMMUNOL.175.7.4355 16177076

[173] Woon HG Braun A Li J Smith C Edwards J Sierro F. Compartmentalization of total and virus-specific tissue-resident memory CD8+ T cells in human lymphoid organs. PloS Pathog (2016) 12:e1005799. doi: 10.1371/JOURNAL.PPAT.1005799 27540722 PMC4991796

[174] Gordon CL Miron M Thome JJC Matsuoka N Weiner J Rak MA. Tissue reservoirs of antiviral T cell immunity in persistent human CMV infection. J Exp Med (2017) 214:651–67. doi: 10.1084/JEM.20160758 PMC5339671 28130404

[175] Pallett LJ Davies J Colbeck EJ Robertson F Hansi N Easom NJW. IL-2high tissue-resident T cells in the human liver: Sentinels for hepatotropic infection. J Exp Med (2017) 214:1567–80. doi: 10.1084/JEM.20162115 PMC5461007 28526759

[176] Zhu J Hladik F Woodward A Klock A Peng T Johnston C. Persistence of HIV-1 receptor–positive cells after HSV-2 reactivation is a potential mechanism for increased HIV-1 acquisition. Nat Med (2009) 15:886–92. doi: 10.1038/nm.2006 PMC2723183 19648930

[177] Schiffer JT Swan DA Prlic M Lund JM. Herpes simplex virus-2 dynamics as a probe to measure the extremely rapid and spatially localized tissue-resident T-cell response. Immunol Rev (2018) 285:113–33. doi: 10.1111/IMR.12672 PMC6752889 30129205

[178] Shin H Kumamoto Y Gopinath S Iwasaki A. CD301b+ dendritic cells stimulate tissue-resident memory CD8+ T cells to protect against genital HSV-2. Nat Commun (2016) 7:13346. doi: 10.1038/ncomms13346 27827367 PMC5105190

[179] Mackay LK Stock AT Ma JZ Jones CM Kent SJ Mueller SN. Long-lived epithelial immunity by tissue-resident memory T (TRM) cells in the absence of persisting local antigen presentation. Proc Natl Acad Sci U.S.A. (2012) 109:7037–42. doi: 10.1073/PNAS.1202288109 PMC3344960 22509047

[180] MacKay LK Rahimpour A Ma JZ Collins N Stock AT Hafon ML. The developmental pathway for CD103+CD8+ tissue-resident memory T cells of skin. Nat Immunol (2013) 14:1294–301. doi: 10.1038/ni.2744 24162776

[181] Hansson GC. Role of mucus layers in gut infection and inflammation. Curr Opin Microbiol (2012) 15:57–62. doi: 10.1016/J.MIB.2011.11.002 22177113 PMC3716454

[182] MacPherson AJ McCoy KD Johansen FE Brandtzaeg P. The immune geography of IgA induction and function. Mucosal Immunol (2008) 1:11–22. doi: 10.1038/mi.2007.6 19079156

[183] Jakobsson HE Rodríguez-Piñeiro AM Schütte A Ermund A Boysen P Bemark M. The composition of the gut microbiota shapes the colon mucus barrier. EMBO Rep (2015) 16:164–77. doi: 10.15252/EMBR.201439263 PMC4328744 25525071

[184] Johansson MEV Sjövall H Hansson GC. The gastrointestinal mucus system in health and disease. Nat Rev Gastroenterol Hepatol (2013) 10:352–61. doi: 10.1038/nrgastro.2013.35 PMC3758667 23478383

[185] Mowat AM Agace WW. Regional specialization within the intestinal immune system. Nat Rev Immunol (2014) 14:667–85. doi: 10.1038/nri3738 25234148

[186] Bowcutt R Forman R Glymenaki M Carding SR Else KJ Cruickshank SM. Heterogeneity across the murine small and large intestine. World J Gastroenterol (2014) 20:15216–32. doi: 10.3748/WJG.V20.I41.15216 PMC4223255 25386070

[187] Agace WW McCoy KD. Regionalized development and maintenance of the intestinal adaptive immune landscape. Immunity (2017) 46:532–48. doi: 10.1016/J.IMMUNI.2017.04.004 28423335

[188] Ghosh D Porter E Shen B Lee SK Wilk D Drazba J. Paneth cell trypsin is the processing enzyme for human defensin-5. Nat Immunol (2002) 3:583–90. doi: 10.1038/ni797 12021776

[189] Porter EM Bevins CL Ghosh D Ganz T. The multifaceted paneth cell. Cell Mol Life Sci (2002) 59:156–70. doi: 10.1007/S00018-002-8412-Z PMC11337504 11846026

[190] McDole JR Wheeler LW McDonald KG Wang B Konjufca V Knoop KA. Goblet cells deliver luminal antigen to CD103+ dendritic cells in the small intestine. Nature (2012) 483:345–9. doi: 10.1038/nature10863 PMC3313460 22422267

[191] Shan M Gentile M Yeiser JR Walland AC Bornstein VU Chen K. Mucus enhances gut homeostasis and oral tolerance by delivering immunoregulatory signals. Science (2013) 342:447–53. doi: 10.1126/SCIENCE.1237910 PMC4005805 24072822

[192] Lelouard H Fallet M de Bovis B Méresse S Gorvel J. Peyer’s patch dendritic cells sample antigens by extending dendrites through m cell-specific transcellular pores. Gastroenterology (2012) 142:592–601.e3. doi: 10.1053/J.GASTRO.2011.11.039 22155637

[193] Hase K Kawano K Nochi T Pontes GS Fukuda S Ebisawa M. Uptake through glycoprotein 2 of FimH+ bacteria by m cells initiates mucosal immune response. Nature (2009) 462:226–30. doi: 10.1038/nature08529 19907495

[194] Niess JH Brand S Gu X Landsman L Jung S McCormick BA. CX3CR1-mediated dendritic cell access to the intestinal lumen and bacterial clearance. Science (2005) 307:254–8. doi: 10.1126/science.1102901 15653504

[195] MacPherson AJ Uhr T. Induction of protective IgA by intestinal dendritic cells carrying commensal bacteria. Science (2004) 303:1662–5. doi: 10.1126/SCIENCE.1091334 15016999

[196] Peterson DA McNulty NP Guruge JL Gordon JI. IgA response to symbiotic bacteria as a mediator of gut homeostasis. Cell Host Microbe (2007) 2:328–39. doi: 10.1016/J.CHOM.2007.09.013 18005754

[197] Farache J Koren I Milo I Gurevich I Kim KW Zigmond E. Luminal bacteria recruit CD103+ dendritic cells into the intestinal epithelium to sample bacterial antigens for presentation. Immunity (2013) 38:581–95. doi: 10.1016/J.IMMUNI.2013.01.009 PMC4115273 23395676

[198] Medina-Contreras O Geem D Laur O Williams IR Lira SA Nusrat A. CX3CR1 regulates intestinal macrophage homeostasis, bacterial translocation, and colitogenic Th17 responses in mice. J Clin Invest (2011) 121:4787–95. doi: 10.1172/JCI59150 PMC3226003 22045567

[199] Panea C Farkas AM Goto Y Abdollahi-Roodsaz S Lee C Koscso B. Intestinal monocyte-derived macrophages control commensal- specific Th17 responses. Cell Rep (2015) 12:1314–24. doi: 10.1016/j.celrep.2015.07.040 PMC4567384 26279572

[200] Persson EK Uronen-Hansson H Semmrich M Rivollier A Hägerbrand K Marsal J. IRF4 transcription-Factor-Dependent CD103+CD11b+ dendritic cells drive mucosal T helper 17 cell differentiation. Immunity (2013) 38:958–69. doi: 10.1016/J.IMMUNI.2013.03.009 23664832

[201] Jaensson E Uronen-Hansson H Pabst O Eksteen B Tian J Coombes JL. Small intestinal CD103+ dendritic cells display unique functional properties that are conserved between mice and humans. J Exp Med (2008) 205:2139–49. doi: 10.1084/JEM.20080414 PMC2526207 18710932

[202] Scott CL Bain CC Wright PB Sichien D Kotarsky K Persson EK. CCR2+CD103– intestinal dendritic cells develop from DC-committed precursors and induce interleukin-17 production by T cells. Mucosal Immunol (2014) 8:327–39. doi: 10.1038/mi.2014.70 PMC4270738 25138666

[203] Iwata M Hirakiyama A Eshima Y Kagechika H Kato C Song S. Retinoic acid imprints gut-homing specificity on T cells. Immunity (2004) 21:527–38. doi: 10.1016/j.immuni.2004.08.011 15485630

[204] Johansson-Lindbom B Svensson M Pabst O Palmqvist C Marquez G Förster R. Functional specialization of gut CD103+ dendritic cells in the regulation of tissue-selective T cell homing. J Exp Med (2005) 202:1063–73. doi: 10.1084/jem.20051100 PMC2213212 16216890

[205] Johansson-Lindbom B Svensson M Wurbel MA Malissen B Márquez G Agace W. Selective generation of gut tropic T cells in gut-associated lymphoid tissue (GALT): requirement for GALT dendritic cells and adjuvant. J Exp Med (2003) 198:963–9. doi: 10.1084/jem.20031244 PMC2194196 12963696

[206] Mora JR Bono MR Manjunath N Weninger W Cavanagh LL Rosemblatt M. Selective imprinting of gut-homing T cells by peyer’s patch dendritic cells. Nature (2003) 424:88–93. doi: 10.1038/nature01726 12840763

[207] Stagg AJ Kamm MA Knight SC. Intestinal dendritic cells increase T cell expression of alpha4beta7 integrin. Eur J Immunol (2002) 32:1445–54. doi: 10.1002/1521-4141(200205)32:5<1445::AID-IMMU1445>3.0.CO;2-E 11981833

[208] Sanders T McCarthy N Giles E Davidson K Haltalli M Hazell S. Increased production of retinoic acid by intestinal macrophages contributes to their inflammatory phenotype in patients with crohn’s disease. Gastroenterology (2014) 146:1278–88. doi: 10.1053/j.gastro.2014.01.057 24503130

[209] Hammerschmidt S Ahrendt M Bode U Wahl B Kremmer E Förster R. Stromal mesenteric lymph node cells are essential for the generation of gut-homing T cells *in vivo* . J Exp Med (2008) 205:2483–90. doi: 10.1084/jem.20080039 PMC2571923 18852290

[210] Jansen M Eestermans IL Kraal G Jochen Huehn RE Förster R Marel W. Lymph node stromal cells support dendritic cell-induced gut-homing of T cells. J Immunol (2022) 183:6395–402. doi: 10.4049/jimmunol.0900311 19841174

[211] Selby WS Janossy G Jewell DP. Immunohistological characterisation of intraepithelial lymphocytes of the human gastrointestinal tract. Gut (1981) 22:169–76. doi: 10.1136/GUT.22.3.169 PMC1419496 7014391

[212] Lundqvist C Baranov V Hammarström S Athlin L Hammarström ML. Intra-epithelial lymphocytes. evidence for regional specialization and extrathymic T cell maturation in the human gut epithelium. Int Immunol (1995) 7:1473–87. doi: 10.1093/INTIMM/7.9.1473 7495755

[213] Jabri B Ebert E. Human CD8+ intraepithelial lymphocytes: A unique model to study the regulation of effector cytotoxic T lymphocytes in tissue. Immunol Rev (2007) 215:202–14. doi: 10.1111/J.1600-065X.2006.00481.X 17291290

[214] Ivanov II McKenzie BS Zhou L Tadokoro CE Lepelley A Lafaille JJ. The orphan nuclear receptor RORγt directs the differentiation program of proinflammatory IL-17+ T helper cells. Cell (2006) 126:1121–33. doi: 10.1016/J.CELL.2006.07.035 16990136

[215] Maynard CL Harrington LE Janowski KM Oliver JR Zindl CL Rudensky AY. Regulatory T cells expressing interleukin 10 develop from Foxp3+ and Foxp3- precursor cells in the absence of interleukin 10. Nat Immunol (2007) 8:931–41. doi: 10.1038/NI1504 17694059

[216] Sathaliyawala T Kubota M Yudanin N Turner D Camp P Thome JJ. Distribution and compartmentalization of human circulating and tissue-resident memory T cell subsets. Immunity (2013) 38:187–97. doi: 10.1016/j.immuni.2012.09.020 PMC3557604 23260195

[217] Veenbergen S Samsom JN. Maintenance of small intestinal and colonic tolerance by IL-10-producing regulatory T cell subsets. Curr Opin Immunol (2012) 24:269–76. doi: 10.1016/J.COI.2012.03.004 22503960

[218] Bartolomé-Casado R Landsverk OJB Chauhan SK Richter L Phung D Greiff V. Resident memory CD8 T cells persist for years in human small intestine. J Exp Med (2019) 216:2412–26. doi: 10.1084/JEM.20190414 PMC6781004 31337737

[219] Bergsbaken T Bevan MJ. Proinflammatory microenvironments within the intestine regulate the differentiation of tissue-resident CD8+ T cells responding to infection. Nat Immunol (2015) 16:406–14. doi: 10.1038/ni.3108 PMC4368475 25706747

[220] Beura L Fares-Frederickson N Steinert EM Scott MC Thompson EA Fraser KA. CD4+ resident memory T cells dominate immunosurveillance and orchestrate local recall responses. J Exp Med (2019) 216:1214–29. doi: 10.1084/jem.20181365 PMC6504216 30923043

[221] Romagnoli P Fu H Qiu Z Khairallah C Pham QM Puddington L. Differentiation of distinct long-lived memory CD4 T cells in intestinal tissues after oral listeria monocytogenes infection. Mucosal Immunol (2017) 10:520–30. doi: 10.1038/mi.2016.66 PMC5272904 27461178

[222] Kurioka A Cosgrove C Simoni Y van Wilgenburg B Geremia A Björkander S. CD161 defines a functionally distinct subset of pro-inflammatory natural killer cells. Front Immunol (2018) 9:486. doi: 10.3389/FIMMU.2018.00486 29686665 PMC5900032

[223] Fergusson JR Hühn MH Swadling L Walker LJ Kurioka A Llibre A. CD161intCD8+ T cells: a novel population of highly functional, memory CD8+ T cells enriched within the gut. Mucosal Immunol (2015) 9:401–13. doi: 10.1038/mi.2015.69 PMC4732939 26220166

[224] Kumar BV Ma W Miron M Granot T Guyer RS Carpenter DJ. Human tissue-resident memory T cells are defined by core transcriptional and functional signatures in lymphoid and mucosal sites. Cell Rep (2017) 20:2921–34. doi: 10.1016/J.CELREP.2017.08.078 PMC5646692 28930685

[225] Bartolomé-Casado R Landsverk OJB Chauhan SK Sætre F Hagen KT Yaqub S. CD4+ T cells persist for years in the human small intestine and display a TH1 cytokine profile. Mucosal Immunol (2020) 14:402–10. doi: 10.1038/s41385-020-0315-5 32572129

[226] Thompson E Mitchell J Beura LK Torres DJ Mrass P Pierson MJ. Interstitial migration of CD8αβ T cells in the small intestine is dynamic and is dictated by environmental cues. Cell Rep (2019) 26:2859–67. doi: 10.1016/j.celrep.2019.02.034 PMC6754515 30865878

[227] Sheridan B Pham Q Lee Y Cauley L Puddington L Lefrancois L. Oral infection drives a distinct population of intestinal resident memory CD8+ T cells with enhanced protective function. Immunity (2014) 40:747–57. doi: 10.1016/j.immuni.2014.03.007 PMC4045016 24792910

[228] Bergsbaken T Bevan M Fink PJ. Local inflammatory cues regulate differentiation and persistence of CD8+ tissue-resident memory T cells. Cell Rep (2017) 19:114–24. doi: 10.1016/j.celrep.2017.03.031 PMC5444811 28380351

[229] Herndler-Brandstetter D Ishigame H Shinnakasu R Plajer V Stecher C Zhao J. KLRG1+ effector CD8+ T cells lose KLRG1, differentiate into all memory T cell lineages, and convey enhanced protective immunity. Immunity (2018) 48:716–29. doi: 10.1016/j.immuni.2018.03.015 PMC6465538 29625895

[230] Schenkel JM Fraser KA Casey KA Beura LK Pauken KE Vezys V. IL-15–independent maintenance of tissue-resident and boosted effector memory CD8 T cells. J Immunol (2016) 196:3920–6. doi: 10.4049/jimmunol.1502337 PMC5145194 27001957

[231] Lyu Y Zhou Y Shen J. An overview of tissue-resident memory T cells in the intestine: From physiological functions to pathological mechanisms. Front Immunol (2022) 0:912393. doi: 10.3389/FIMMU.2022.912393 PMC9192946 35711464

[232] Paap EM Müller TM Sommer K Neurath MF Zundler S. Total recall: Intestinal TRM cells in health and disease. Front Immunol (2021) 11:623072. doi: 10.3389/FIMMU.2020.623072 33542725 PMC7851044

[233] Turnbull EL Yrlid U Jenkins CD MacPherson GG. Intestinal dendritic cell subsets: differential effects of systemic TLR4 stimulation on migratory fate and activation *in vivo* . J Immunol (2005) 174:1374–84. doi: 10.4049/jimmunol.174.3.1374 15661895

[234] Yrlid U Milling SW Miller JL Cartland S Jenkins CD MacPherson GG. Regulation of intestinal dendritic cell migration and activation by plasmacytoid dendritic cells, TNF-α and type 1 IFNs after feeding a TLR7/8 ligand. J Immunol (2006) 176:5205–12. doi: 10.4049/jimmunol.176.9.5205 16621985

[235] Huang G Wang Y Chi H. Control of T cell fates and immune tolerance by p38α signaling in mucosal CD103+ dendritic cells. J Immunol (2013) 191:650–9. doi: 10.4049/jimmunol.1300398 PMC3702677 23752611

[236] Kinnebrew MA Buffie CG Diehl GE Zenewicz LA Leiner I Hohl TM. Interleukin 23 production by intestinal CD103 +CD11b + dendritic cells in response to bacterial flagellin enhances mucosal innate immune defense. Immunity (2012) 36:276–87. doi: 10.1016/J.IMMUNI.2011.12.011 PMC3288454 22306017

[237] Ivanov II Atarashi K Manel N Brodie EL Shima T Karaoz U. Induction of intestinal Th17 cells by segmented filamentous bacteria. Cell (2009) 139:485–98. doi: 10.1016/J.CELL.2009.09.033 PMC2796826 19836068

[238] Goto Y Panea C Nakato G Cebula A Lee C Diez MG. Segmented filamentous bacteria antigens presented by intestinal dendritic cells drive mucosal Th17 cell differentiation. Immunity (2014) 40:594–607. doi: 10.1016/J.IMMUNI.2014.03.005 24684957 PMC4084624

[239] Salzman NH Hung K Haribhai D Chu H Karlsson-Sjöberg J Amir E. Enteric defensins are essential regulators of intestinal microbial ecology. Nat Immunol (2009) 11:76–82. doi: 10.1038/ni.1825 19855381 PMC2795796

[240] Powrie F Mason D. OX-22high CD4+ T cells induce wasting disease with multiple organ pathology: prevention by the OX-22low subset. J Exp Med (1990) 172:1701–8. doi: 10.1084/jem.172.6.1701 PMC2188779 2258700

[241] Gambineri E Torgerson TR Ochs HD. Immune dysregulation, polyendocrinopathy, enteropathy, and X-linked inheritance (IPEX), a syndrome of systemic autoimmunity caused by mutations of FOXP3, a critical regulator of T-cell homeostasis. Curr Opin Rheumatol (2003) 15:430–5. doi: 10.1097/00002281-200307000-00010 12819471

[242] Powrie F Leach MW Mauze S Caddle LB Coffman RL. Phenotypically distinct subsets of CD4+ T cells induce or protect from chronic intestinal inflammation in c. b-17 scid mice. Int Immunol (1993) 5:1461–71. doi: 10.1093/intimm/5.11.1461 7903159

[243] Josefowicz S Niec R Kim H Treuting P Chinen T Zheng Y. Extrathymically generated regulatory T cells control mucosal TH2 inflammation. Nature (2012) 482:395–9. doi: 10.1038/nature10772 PMC3485072 22318520

[244] Campbell C Dikiy S Bhattarai S Chinen T Matheis F Calafiore M. Extrathymically generated regulatory T cells establish a niche for intestinal border-dwelling bacteria and affect physiologic metabolite balance. Immunity (2018) 48:1245–1257.e9. doi: 10.1016/j.immuni.2018.04.013 29858010 PMC6260932

[245] Kim KS Hong SW Han D Yi J Jung J Yang BG. Dietary antigens limit mucosal immunity by inducing regulatory T cells in the small intestine. Science (2016) 351:858–63. doi: 10.1126/SCIENCE.AAC5560 26822607

[246] Sefik E Geva-Zatorsky N Oh S Konnikova L Zemmour D McGuire AM. Individual intestinal symbionts induce a distinct population of RORγ+ regulatory T cells. Science (2015) 349:993–7. doi: 10.1126/SCIENCE.AAA9420 PMC4700932 26272906

[247] Ohnmacht C Park JH Cording S Wing JB Atarashi K Obata Y. The microbiota regulates type 2 immunity through RORγt+ T cells. Science (2015) 349:989–93. doi: 10.1126/SCIENCE.AAC4263 26160380

[248] Yang B Hagemann S Mamareli P Lauer U Hoffmann U Beckstette M. Foxp3(+) T cells expressing RORγt represent a stable regulatory T-cell effector lineage with enhanced suppressive capacity during intestinal inflammation. Mucosal Immunol (2016) 9:444–57. doi: 10.1038/mi.2015.74 26307665

[249] Wohlfert E Grainger J Bouladoux N Konkel JE Oldenhove G Ribeiro CH. GATA3 controls Foxp3+ regulatory T cell fate during inflammation in mice. J Clin Invest (2011) 121:4503–15. doi: 10.1172/JCI57456 PMC3204837 21965331

[250] Schiering C Krausgruber T Chomka A Fröhlich A Adelmann K Wohlfert E. The alarmin IL-33 promotes regulatory T-cell function in the intestine. Nature (2014) 513:564–8. doi: 10.1038/nature13577 PMC4339042 25043027

[251] Konkel J Chen W. Balancing acts: the role of TGF-β in the mucosal immune system. Trends Mol Med (2011) 17:668–76. doi: 10.1016/j.molmed.2011.07.002 PMC3205325 21890412

[252] Worthington J Czajkowska B Melton A Travis MA. Intestinal dendritic cells specialize to activate transforming growth factor-β and induce Foxp3+ regulatory T cells *via* integrin αvβ8. Gastroenterology (2011) 141:1802–12. doi: 10.1053/j.gastro.2011.06.057 PMC3507624 21723222

[253] Konkel J Zhang D Zanvit P Chia C Zangarle-Murray T Jin W. Transforming growth factor-β signaling in regulatory T cells controls T helper-17 cells and tissue-specific immune responses. Immunity (2017) 46:660–74. doi: 10.1016/j.immuni.2017.03.015 PMC12230991 28423340

[254] Coombes J Siddiqui KR Arancibia-Cárcamo CV Hall J Sun CM Belkaid Y. A functionally specialized population of mucosal CD103+ DCs induces Foxp3+ regulatory T cells *via* a TGF-beta and retinoic acid-dependent mechanism. J Exp Med (2007) 204:1757–64. doi: 10.1084/jem.20070590 PMC2118683 17620361

[255] Sun CM Hall JA Blank RB Bouladoux N Oukka M Mora JR. Small intestine lamina propria dendritic cells promote *de novo* generation of Foxp3 T reg cells *via* retinoic acid. J Exp Med (2007) 204:1775–85. doi: 10.1084/jem.20070602 PMC2118682 17620362

[256] Mucida D Park Y Kim G Turovskaya O Scott I Kronenberg M. Reciprocal TH17 and regulatory T cell differentiation mediated by retinoic acid. Science (2007) 317:256–60. doi: 10.1126/SCIENCE.1145697 17569825

[257] Annacker O Coombes JL Malmstrom V Uhlig HH Bourne T Johansson-Lindbom B. Essential role for CD103 in the T cell-mediated regulation of experimental colitis. J Exp Med (2005) 202:1051–61. doi: 10.1084/jem.20040662 PMC2213206 16216886

[258] Travis MA Reizis B Melton AC Masteller E Tang Q Proctor JM. Loss of integrin alpha(v)beta8 on dendritic cells causes autoimmunity and colitis in mice. Nature (2007) 449:361–5. doi: 10.1038/nature06110 PMC2670239 17694047

[259] Povoleri GAM Nova-Lamperti E Scottà C Fanelli G Chen YC Becker PD. Human retinoic acid-regulated CD161+ regulatory T cells support wound repair in intestinal mucosa. Nat Immunol (2018) 19:1403–14. doi: 10.1038/s41590-018-0230-z PMC6474659 30397350

[260] Round JL Mazmanian SK. Inducible Foxp3+ regulatory T-cell development by a commensal bacterium of the intestinal microbiota. Proc Natl Acad Sci U.S.A. (2010) 107:12204–9. doi: 10.1073/PNAS.0909122107 PMC2901479 20566854

[261] Edelblum KL Shen L Weber CR Marchiando AM Clay BS Wang Y. Dynamic migration of γδ intraepithelial lymphocytes requires occludin. Proc Natl Acad Sci U.S.A. (2012) 109:7097–102. doi: 10.1073/PNAS.1112519109 PMC3345021 22511722

[262] Di Marco Barros R Roberts NA Dart RJ Vantourout P Jandke A Nussbaumer O. Epithelia use butyrophilin-like molecules to shape organ-specific γδ T cell compartments. Cell (2016) 167:203–218.e17. doi: 10.1016/j.cell.2016.08.030 27641500 PMC5037318

[263] Boismenu R Havran WL. Modulation of epithelial cell growth by intraepithelial γδ T cells. Science (1994) 266:1253–5. doi: 10.1126/science.7973709 7973709

[264] Komano H Fujiura Y Kawaguchi M Matsumoto S Hashimoto Y Obana S. Homeostatic regulation of intestinal epithelia by intraepithelial γδ T cells. Proc Natl Acad Sci U.S.A. (1995) 92:6147–51. doi: 10.1073/PNAS.92.13.6147 PMC41659 7597094

[265] Dalton JE Cruickshank SM Egan CE Mears R Newton DJ Andrew EM. Intraepithelial gammadelta+ lymphocytes maintain the integrity of intestinal epithelial tight junctions in response to infection. Gastroenterology (2006) 131:818–29. doi: 10.1053/j.gastro.2006.06.003 16952551

[266] Bhagat G Naiyer AJ Shah JG Harper J Jabri B Wang TC. Small intestinal CD8+TCRgammadelta+NKG2A+ intraepithelial lymphocytes have attributes of regulatory cells in patients with celiac disease. J Clin Invest (2008) 118:281–93. doi: 10.1172/JCI30989 PMC2117760 18064301

[267] Danese S Fiocchi C. Etiopathogenesis of inflammatory bowel diseases. World J Gastroenterol (2006) 12:4807–12. doi: 10.3748/wjg.v12.i30.4807 PMC4087613 16937461

[268] Matricon J Barnich N Ardid D. Immunopathogenesis of inflammatory bowel disease. Self Nonself (2010) 1:299–309. doi: 10.4161/self.1.4.13560 21487504 PMC3062384

[269] Hart AL Al-Hassi HO Rigby RJ Bell SJ Emmanuel AV Knight SC. Characteristics of intestinal dendritic cells in inflammatory bowel diseases. Gastroenterology (2005) 129:50–65. doi: 10.1053/J.GASTRO.2005.05.013 16012934

[270] Sakuraba A Sato T Kamada N Kitazume M Sugita A Hibi T. Th1/Th17 immune response is induced by mesenteric lymph node dendritic cells in crohn’s disease. Gastroenterology (2009) 137:1736–45. doi: 10.1053/J.GASTRO.2009.07.049 19632232

[271] Magnusson MK Brynjólfsson SF Dige A Uronen-Hansson H Börjesson LG Bengtsson JL. Macrophage and dendritic cell subsets in IBD: ALDH+ cells are reduced in colon tissue of patients with ulcerative colitis regardless of inflammation. Mucosal Immunol (2015) 9:171–82. doi: 10.1038/mi.2015.48 PMC4683124 26080709

[272] Salim SY Silva MA Keita ÅV Larsson M Andersson P Magnusson KE. CD83+CCR7– dendritic cells accumulate in the subepithelial dome and internalize translocated escherichia coli HB101 in the peyer’s patches of ileal crohn’s disease. Am J Pathol (2009) 174:82–90. doi: 10.2353/AJPATH.2009.080273 19095953 PMC2631321

[273] Peyrin-Biroulet L Chamaillard M Gonzalez F Beclin E Decourcelle C Antunes L. Mesenteric fat in crohn’s disease: A pathogenetic hallmark or an innocent bystander? Gut (2007) 56:577–83. doi: 10.1136/GUT.2005.082925 PMC1856873 16956921

[274] Al-Hassi HO Bernardo D Murugananthan AU Mann ER English NR Jones A. A mechanistic role for leptin in human dendritic cell migration: Differences between ileum and colon in health and crohn’s disease. Mucosal Immunol (2012) 6:751–61. doi: 10.1038/mi.2012.113 PMC3684777 23168838

[275] Zundler S Becker E Spocinska M Slawik M Parga-Vidal L Stark R. Hobit- and blimp-1-driven CD4+ tissue-resident memory T cells control chronic intestinal inflammation. Nat Immunol (2019) 20:288–300. doi: 10.1038/s41590-018-0298-5 30692620

[276] Bishu S el Zaatari M Hayashi A Hou G Bowers N Kinnucan J. CD4+ tissue-resident memory T cells expand and are a major source of mucosal tumour necrosis factor α in active crohn’s disease. J Crohns Colitis (2019) 13:905–15. doi: 10.1093/ECCO-JCC/JJZ010 PMC6939878 30715262

[277] Bottois H Ngollo M Hammoudi N Courau T Bonnereau J Chardiny V. KLRG1 and CD103 expressions define distinct intestinal tissue-resident memory CD8 T cell subsets modulated in crohn’s disease. Front Immunol (2020) 11:896. doi: 10.3389/FIMMU.2020.00896 32477365 PMC7235448

[278] Boland BS He Z Tsai MS Olvera JG Omilusik KD Duong HG. Heterogeneity and clonal relationships of adaptive immune cells in ulcerative colitis revealed by single-cell analyses. Sci Immunol (2020) 5:eabb4432. doi: 10.1126/SCIIMMUNOL.ABB4432 32826341 PMC7733868

[279] Corridoni D Antanaviciute A Gupta T Fawkner-Corbett D Aulicino A Jagielowicz M. Single-cell atlas of colonic CD8+ T cells in ulcerative colitis. Nat Med (2020) 26:1480–90. doi: 10.1038/s41591-020-1003-4 32747828

[280] van Unen V Ouboter LF Li N Schreurs M Abdelaal T Kooy-Winkelaar Y. Identification of a disease-associated network of intestinal immune cells in treatment-naive inflammatory bowel disease. Front Immunol (2022) 0:893803. doi: 10.3389/FIMMU.2022.893803 PMC9260579 35812429

[281] Noble A Durant L Hoyles L McCartney AL Man R Segal J. Deficient resident memory T cell and CD8 T cell response to commensals in inflammatory bowel disease. J Crohns Colitis (2020) 14:525–37. doi: 10.1093/ECCO-JCC/JJZ175 PMC7242004 31665283

[282] Roosenboom B Wahab PJ Smids C Groenen MJM van Koolwijk E van Lochem EG. Intestinal CD103+CD4+ and CD103+CD8+ T-cell subsets in the gut of inflammatory bowel disease patients at diagnosis and during follow-up. Inflammation Bowel Dis (2019) 25:1497–509. doi: 10.1093/IBD/IZZ049 PMC6701511 30918941

[283] Estimating the burden of enteric disease . Available at: https://www.who.int/teams/immunization-vaccines-and-biologicals/product-and-delivery-research/burden-of-enteric-diseases (Accessed 23. August 2022).

[284] Donnenberg MS Narayanan S. How to diagnose a foodborne illness. Infect Dis Clin North Am (2013) 27:535–54. doi: 10.1016/J.IDC.2013.05.001 24011829

[285] Navaneethan U Giannella RA. Mechanisms of infectious diarrhea. Nat Clin Pract Gastroenterol Hepatol (2008) 5:637–47. doi: 10.1038/ncpgasthep1264 18813221

[286] Anderson DJ Murdoch DR Sexton DJ Reller LB Stout JE Cabell CH. Risk factors for infective endocarditis in patients with enterococcal bacteremia: A case-control study. Infection (2004) 32:72–7. doi: 10.1007/s15010-004-2036-1 15057570

[287] Kelly CP Pothoulakis C LaMont JT. Clostridium difficile colitis. N Engl J Med (1994) 330:257–62. doi: 10.1056/NEJM199401273300406 8043060

[288] Hayashi F Smith KD Ozinsky A Hawn TR Yi EC Goodlett DR. The innate immune response to bacterial flagellin is mediated by toll-like receptor 5. Nature (2001) 410:1099–103. doi: 10.1038/35074106 11323673

[289] Uematsu S Akira S. Immune responses of TLR5+ lamina propria dendritic cells in enterobacterial infection. J Gastroenterol (2009) 44:803–11. doi: 10.1007/S00535-009-0094-Y 19547909

[290] Godinez I Keestra AM Spees A Bäumler AJ. The IL-23 axis in salmonella gastroenteritis. Cell Microbiol (2011) 13:1639–47. doi: 10.1111/J.1462-5822.2011.01637.X 21740501

[291] Mangan PR Harrington LE O’Quinn DB Helms WS Bullard DC Elson CO. Transforming growth factor-β induces development of the TH17 lineage. Nature (2006) 441:231–4. doi: 10.1038/nature04754 16648837

[292] Edwards LA Nistala K Mills DC Stephenson HN Zilbauer M Wren BW. Delineation of the innate and adaptive T-cell immune outcome in the human host in response to campylobacter jejuni infection. PloS One (2010) 5:e15398. doi: 10.1371/JOURNAL.PONE.0015398 21085698 PMC2976761

[293] Awasthi A Riol-Blanco L Jäger A Korn T Pot C Galileos G. Cutting edge: IL-23 receptor GFP reporter mice reveal distinct populations of IL-17-Producing cells. J Immunol (2009) 182:5904–8. doi: 10.4049/JIMMUNOL.0900732 PMC2702203 19414740

[294] Zhou L Ivanov II Spolski R Min R Shenderov K Egawa T. IL-6 programs TH-17 cell differentiation by promoting sequential engagement of the IL-21 and IL-23 pathways. Nat Immunol (2007) 8:967–74. doi: 10.1038/ni1488 17581537

[295] Geddes K Rubino SJ Magalhaes JG Streutker C le Bourhis L Cho JH. Identification of an innate T helper type 17 response to intestinal bacterial pathogens. Nat Med (2011) 17:837–44. doi: 10.1038/nm.2391 21666695

[296] Raffatellu M Santos RL Verhoeven DE George MD Wilson RP Winter SE. Simian immunodeficiency virus–induced mucosal interleukin-17 deficiency promotes salmonella dissemination from the gut. Nat Med (2008) 14:421–8. doi: 10.1038/nm1743 PMC2901863 18376406

[297] Ishigame H Kakuta S Nagai T Kadoki M Nambu A Komiyama Y. Differential roles of interleukin-17A and -17F in host defense against mucoepithelial bacterial infection and allergic responses. Immunity (2009) 30:108–19. doi: 10.1016/J.IMMUNI.2008.11.009 19144317

[298] Kuchta A Rahman T Sennott EL Bhuyian TR Uddin T Rashu R. Vibrio cholerae O1 infection induces proinflammatory CD4 + T-cell responses in blood and intestinal mucosa of infected humans. Clin Vaccine Immunol (2011) 18:1371–7. doi: 10.1128/CVI.05088-11 PMC3147337 21697339

[299] Behnsen J Jellbauer S Wong CP Edwards RA George MD Ouyang W. The cytokine IL-22 promotes pathogen colonization by suppressing related commensal bacteria. Immunity (2014) 40:262–73. doi: 10.1016/J.IMMUNI.2014.01.003 PMC3964146 24508234

[300] Zheng Y Valdez PA Danilenko DM Hu Y Sa SM Gong Q. Interleukin-22 mediates early host defense against attaching and effacing bacterial pathogens. Nat Med (2008) 14:282–9. doi: 10.1038/nm1720 18264109

[301] Ahlfors H Morrison PJ Duarte JH Li Y Biro J Tolaini M. IL-22 fate reporter reveals origin and control of IL-22 production in homeostasis and infection. J Immunol (2014) 193:4602–13. doi: 10.4049/JIMMUNOL.1401244 PMC4201943 25261485

[302] Schulke L Manconi F Markham R Fraser IS. Endometrial dendritic cell populations during the normal menstrual cycle. Hum Reprod (2008) 23:1574–80. doi: 10.1093/humrep/den030 18285323

[303] Agostinis C Mangogna A Bossi F Ricci G Kishore U Bulla R. Uterine immunity and microbiota: A shifting paradigm. Front Immunol (2019) 10:2387. doi: 10.3389/fimmu.2019.02387 31681281 PMC6811518

[304] Flynn L Byrne B Carton J Kelehan P O’Herlihy C O’Farrelly C. Menstrual cycle dependent fluctuations in NK and T-lymphocyte subsets from non-pregnant human endometrium. Am J Reprod Immunol (2000) 43:209–17. doi: 10.1111/j.8755-8920.2000.430405.x 10836250

[305] Bonatz G Hansmann M-L Buchholz F Mettler L Radzun HJ Semm K. Macrophage- and lymphocyte-subtypes in the endometrium during different phases of the ovarian cycle. Int J Gynaecol Obstet (1992) 37:29–36. doi: 10.1016/0020-7292(92)90974-N 1346597

[306] Butts CL Candando KM Warfel J Belyavskaya E Sternberg EM. Progesterone regulation of uterine dendritic cell function in rodents is dependent on the stage of estrous cycle. Mucosal Immunol (2010) 3:496–505. doi: 10.1038/mi.2010.28 20505661

[307] Lee J Ulrich B Cho J Kim HC. Progesterone promotes differentiation of human cord blood fetal T cells into T regulatory cells but suppresses their differentiation into Th17 cells. J Immunol (2011) 187:1778–87. doi: 10.4049/jimmunol.1003919 PMC3155957 21768398

[308] Miyaura H Iwata M. Direct and indirect inhibition of Th1 development by progesterone and glucocorticoids. J Immunol (2002) 168:1087–94. doi: 10.4049/jimmunol.168.3.1087 11801642

[309] Ghosh M Rodriguez-Garcia M Wira CR. The immune system in menopause: Pros and cons of hormone therapy. J Steroid Biochem Mol Biol (2014) 142:171–5. doi: 10.1016/j.jsbmb.2013.09.003 PMC3954964 24041719

[310] Iijima N Thompson JM Iwasaki A. Dendritic cells and macrophages in the genitourinary tract. Mucosal Immunol (2008) 1:451–9. doi: 10.1038/mi.2008.57 PMC2684461 19079212

[311] Duluc D Gannevat J Anguiano E Zurawski S Carley M Boreham M. Functional diversity of human vaginal APC subsets in directing T-cell responses. Mucosal Immunol (2013) 6:626–38. doi: 10.1038/mi.2012.104 PMC3568194 23131784

[312] Duluc D Gannevat J Joo HM Ni L Upchurch K Boreham M. Dendritic cells and vaccine design for sexually-transmitted diseases. Microb Pathog (2013) 58:35–44. doi: 10.1016/j.micpath.2012.11.010 23201532 PMC3596496

[313] Rodriguez-Garcia M Shen Z Barr FD Boesch AW Ackerman ME Kappes JC. Dendritic cells from the human female reproductive tract rapidly capture and respond to HIV. Mucosal Immunol (2017) 10:531–44. doi: 10.1038/mi.2016.72 PMC5332537 27579858

[314] Stary G Olive A Radovic-moreno AF Gondek D Basto PA Perro M. A mucosal vaccine against chlamydia trachomatis generates two waves of protective memory T cells. Science (2015) 348:aaa8205. doi: 10.1126/science.aaa8205.A 26089520 PMC4605428

[315] Collins MK Tay CS Erlebacher A. Dendritic cell entrapment within the pregnant uterus inhibits immune surveillance of the maternal/fetal interface in mice. J Clin Invest (2009) 119:2062–73. doi: 10.1172/JCI38714 PMC2701881 19546507

[316] Garcia-Alonso L Handfield LF Roberts K Nikolakopoulou K Fernando RC Gardner L. Mapping the temporal and spatial dynamics of the human endometrium *in vivo* and *in vitro* . Nat Genet (2021) 53:1698–711. doi: 10.1038/s41588-021-00972-2 PMC8648563 34857954

[317] Woodward Davis AS Vick SC Pattacini L Voullet V Hughes SM Lentz GM. The human memory T cell compartment changes across tissues of the female reproductive tract. Mucosal Immunol (2021) 14:862–72. doi: 10.1007/s00109-020-02028-0 PMC8225572 33953338

[318] Peng T Phasouk K Bossard E Klock A Jin L Laing KJ. Distinct populations of antigen-specific tissue-resident CD8+ T cells in human cervix mucosa. JCI Insight (2021) 6:1–17. doi: 10.1172/jci.insight.149950 PMC8410090 34156975

[319] Koelle DM Dong L Jing L Laing KJ Zhu J Jin L. HSV-2-Specific human female reproductive tract tissue resident memory T cells recognize diverse HSV antigens. Front Immunol (2022) 13:867962. doi: 10.3389/fimmu.2022.867962 35432373 PMC9009524

[320] Ssemaganda A Cholette F Perner M Kambaran C Adhiambo W Wambugu PM. Endocervical regulatory T cells are associated with decreased genital inflammation and lower HIV target cell abundance. Front Immunol (2021) 12:726472. doi: 10.3389/fimmu.2021.726472 34630402 PMC8495419

[321] Robertson SA Care AS Moldenhauer LM. Regulatory T cells in embryo implantation and the immune response to pregnancy. J Clin Invest (2018) 128:4224–35. doi: 10.1172/JCI122182 PMC6159994 30272581

[322] Tsuda S Nakashima A Shima T Saito S. New paradigm in the role of regulatory T cells during pregnancy. Front Immunol (2019) 10:573. doi: 10.3389/fimmu.2019.00573 30972068 PMC6443934

[323] Traxinger BR Richert-Spuhler LE Lund JM. Mucosal tissue regulatory T cells are integral in balancing immunity and tolerance at portals of antigen entry. Mucosal Immunol (2021) 15:398–407. doi: 10.1038/s41385-021-00471-x PMC8628059 34845322

[324] Imarai M Candia E Rodriguez-Tirado C Tognarelli J Pardo M Pérez T. Regulatory T cells are locally induced during intravaginal infection of mice with neisseria gonorrhoeae. Infect Immun (2008) 76:5456–65. doi: 10.1128/IAI.00552-08 PMC2583596 18824531

[325] Cai D Tang Y Yao X. Changes of γδT cell subtypes during pregnancy and their influences in spontaneous abortion. J Reprod Immunol (2019) 131:57–62. doi: 10.1016/j.jri.2019.01.003 30710888

[326] Terzieva A Dimitrova V Djerov L Dimitrova P Zapryanova S Hristova I. Early pregnancy human decidua is enriched with activated, fully differentiated and pro-inflammatory gamma/delta T cells with diverse TCR repertoires. Int J Mol Sci (2019) 20:1–18. doi: 10.3390/ijms20030687 PMC6387174 30764544

[327] Strbo N Alcaide ML Romero L Bolivar H Jones D Podack ER. Loss of intraepithelial endocervical gamma delta (GD) 1 T cells in HIV infected women. Am J Reprod Immunol (2016) 75:134–45. doi: 10.1111/aji.12458 PMC4715976 26666220

[328] Alcaide ML Strbo N Romero L Jones DL Rodriguez VJ Arheart K. Bacterial vaginosis is associated with loss of gamma delta T cells in the female reproductive tract in women in the Miami women interagency HIV study (WIHS): A cross sectional study. PloS One (2016) 11:1–14. doi: 10.1371/journal.pone.0153045 PMC4831836 27078021

[329] Kim JO Cha HR Kim E Kweon MN. Pathological effect of IL-17A-producing TCRγδ+ T cells in mouse genital mucosa against HSV-2 infection. Immunol Lett (2012) 147:34–40. doi: 10.1016/j.imlet.2012.05.006 22698680

[330] Monin L Ushakov DS Arnesen H Bah N Jandke A Muñoz-Ruiz M. γδ T cells compose a developmentally regulated intrauterine population and protect against vaginal candidiasis. Mucosal Immunol (2020) 13:969–81. doi: 10.1038/s41385-020-0305-7 PMC7567646 32472066

[331] Perez-Zsolt D Cantero-Pérez J Erkizia I Benet S Pino M Serra-Peinado C. Dendritic cells from the cervical mucosa capture and transfer HIV-1 *via* siglec-1. Front Immunol (2019) 10:825. doi: 10.3389/fimmu.2019.00825 31114569 PMC6503733

[332] Rodriguez-Garcia M Barr FD Crist SG Fahey JV Wira CR. Phenotype and susceptibility to HIV infection of CD4+ Th17 cells in the human female reproductive tract. Mucosal Immunol (2014) 7:1375–85. doi: 10.1038/mi.2014.26 PMC4205172 24759207

[333] Cantero-Pérez J Grau-Expósito J Serra-Peinado C Rosero DA Luque-Ballesteros L Astorga-Gamaza A. Resident memory T cells are a cellular reservoir for HIV in the cervical mucosa. Nat Commun (2019) 10:4739. doi: 10.1038/s41467-019-12732-2 31628331 PMC6802119

[334] Iyer SS Sabula MJ Mehta CC Haddad LB Brown NL Amara RR. Characteristics of HIV target CD4 T cells collected using different sampling methods from the genital tract of HIV seronegative women. PloS One (2017) 12:e0178193. doi: 10.1371/journal.pone.0178193 28570576 PMC5453484

[335] Saluzzo S Pandey RV Gail LM Dingelmaier-Hovorka R Kleissl L Shaw L. Delayed antiretroviral therapy in HIV-infected individuals leads to irreversible depletion of skin- and mucosa-resident memory T cells. Immunity (2021) 54:2842–2858.e5. doi: 10.1016/j.immuni.2021.10.021 34813775

[336] Caruso MP Falivene J Holgado MP Zurita DH Laufer N Castro C. Impact of HIV-ART on the restoration of Th17 and treg cells in blood and female genital mucosa. Sci Rep (2019) 9:1–16. doi: 10.1038/s41598-019-38547-1 30760809 PMC6374372

[337] Kelly H Chikandiwa A Alemany Vilches L Palefsky JM de Sanjose S Mayaud P. Association of antiretroviral therapy with anal high-risk human papillomavirus, anal intraepithelial neoplasia, and anal cancer in people living with HIV: A systematic review and meta-analysis. Lancet HIV (2020) 7:e262–78. doi: 10.1016/S2352-3018(19)30434-5 32109408

[338] Brickman C Palefsky JM. Human papillomavirus in the HIV-infected host: Epidemiology and pathogenesis in the antiretroviral era. Curr HIV/AIDS Rep (2015) 12:6–15. doi: 10.1007/s11904-014-0254-4 25644977

[339] Mbuya W McHaro R Mhizde J Mnkai J Mahenge A Mwakatima M. Depletion and activation of mucosal CD4 T cells in HIV infected women with HPV associated lesions of the cervix uteri. PloS One (2020) 15:1–17. doi: 10.1371/journal.pone.0240154 PMC7531815 33007050

[340] Cao Y Zhao J Lei Z Shen S Liu C Li D. Local accumulation of FOXP3 + regulatory T cells: Evidence for an immune evasion mechanism in patients with Large condylomata acuminata. J Immunol (2008) 180:7681–6. doi: 10.4049/jimmunol.180.11.7681 18490771

[341] Pahne-Zeppenfeld J Schröer N Walch-Rückheim B Oldak M Gorter A Hegde S. Cervical cancer cell-derived interleukin-6 impairs CCR7-dependent migration of MMP-9-expressing dendritic cells. Int J Cancer (2014) 134:2061–73. doi: 10.1002/ijc.28549 24136650

[342] Le Borgne M Etchart N Goubier A Lira SA Sirard JC van Rooijen N. Dendritic cells rapidly recruited into epithelial tissues *via* CCR6/CCL20 are responsible for CD8^+^ T cell crosspriming *In vivo* . Immunity (2006) 24:191–201. doi: 10.1016/j.immuni.2006.01.005 16473831

[343] Bashaw AA Leggatt GR Chandra J Tuong ZK Frazer IH. Modulation of antigen presenting cell functions during chronic HPV infection. Papillomavirus Res (2017) 4:58–65. doi: 10.1016/j.pvr.2017.08.002 29179871 PMC5883240

[344] Xue JS Wang YL Chen C Zhu XJ Zhu H Hu Y. Effects of Th17 cells and IL-17 in the progression of cervical carcinogenesis with high-risk human papillomavirus infection. Cancer Med (2018) 7:297–306. doi: 10.1002/cam4.1279 29277958 PMC5806118

[345] Komdeur FL Prins TM van de Wall S Plat A Wisman GBA Hollema H. CD103+ tumor-infiltrating lymphocytes are tumor-reactive intraepithelial CD8+ T cells associated with prognostic benefit and therapy response in cervical cancer. Oncoimmunology (2017) 6:1–14. doi: 10.1080/2162402X.2017.1338230 PMC5599086 28932636

[346] Vlcek KR Li W Manam S Zanotti B Nicholson BJ Ramsey KH. The contribution of chlamydia-specific CD8^+^ T cells to upper genital tract pathology. Immunol Cell Biol (2016) 94:208–12. doi: 10.1038/icb.2015.74 PMC4747851 26323581

[347] Gondek DC Olive AJ Stary G Starnbach MN. CD4+ T cells are necessary and sufficient to confer protection against c. trachomatis infection in the murine upper genital tract. J Immunol (2012) 189:2441–9. doi: 10.4049/jimmunol.1103032 PMC3690950 22855710

[348] Perry LL Feilzer K Caldwell HD. Immunity to chlamydia trachomatis is mediated by T helper 1 cells through IFN-gamma-dependent and -independent pathways. J Immunol (1997) 158:3344–52. 9120292

[349] Rixon JA Depew CE McSorley SJ. Th1 cells are dispensable for primary clearance of chlamydia from the female reproductive tract of mice. PloS Pathog (2022) 18:1–20. doi: 10.1371/journal.ppat.1010333 PMC8901068 35196366

[350] Gondek DC Roan NR Starnbach MN. T Cell responses in the absence of IFNγ exacerbate uterine infection with chlamydia trachomatis. J Immunol (2009) 183:1313–9. doi: 10.4049/jimmunol.0900295.T PMC2723820 19561106

[351] Labuda JC Pham OH Depew CE Fong KD Lee B Rixon JA. Circulating immunity protects the female reproductive tract from chlamydia infection. Proc Natl Acad Sci U.S.A. (2021) 118:e2104407118. doi: 10.1073/pnas.2104407118 34001624 PMC8166081

[352] Lijek RS Helble JD Olive AJ Seiger KW Starnbach MN. Pathology after chlamydia trachomatis infection is driven by nonprotective immune cells that are distinct from protective populations. Proc Natl Acad Sci U.S.A. (2018) 115:2216–21. doi: 10.1073/pnas.1711356115 PMC5834673 29440378

[353] Moore-Connors JM Fraser R Halperin SA Wang J. CD4 + CD25 + Foxp3 + regulatory T cells promote Th17 responses and genital tract inflammation upon intracellular chlamydia muridarum infection. J Immunol (2013) 191:3430–9. doi: 10.4049/jimmunol.1301136 23956419

[354] Efremova M Vento-Tormo M Teichmann SA Vento-Tormo R. CellPhoneDB: inferring cell–cell communication from combined expression of multi-subunit ligand–receptor complexes. Nat Protoc (2020) 15:1484–506. doi: 10.1038/s41596-020-0292-x 32103204

[355] Jin S Guerrero-Juarez CF Zhang L Chang I Ramos R Kuan CH. Inference and analysis of cell-cell communication using CellChat. Nat Commun (2021) 12:1–20. doi: 10.1038/s41467-021-21246-9 33597522 PMC7889871

[356] Popescu DM Botting RA Stephenson E Green K Webb S Jardine L. Decoding human fetal liver haematopoiesis. Nature (2019) 574:365–71. doi: 10.1038/s41586-019-1652-y PMC6861135 31597962

[357] Park JE Botting RA Conde CD Popescu DM Lavaert M Kunz DJ. A cell atlas of human thymic development defines T cell repertoire formation. Science (2020) 367:eaay3224. doi: 10.1126/SCIENCE.AAY3224 32079746 PMC7611066

[358] Elmentaite R Kumasaka N Roberts K Fleming A Dann E King HW. Cells of the human intestinal tract mapped across space and time. Nature (2021) 597:250–5. doi: 10.1038/s41586-021-03852-1 PMC8426186 34497389

[359] Penkava F Velasco-Herrera MDC Young MD Yager N Nwosu LN Pratt AG. Single-cell sequencing reveals clonal expansions of pro-inflammatory synovial CD8 T cells expressing tissue-homing receptors in psoriatic arthritis. Nat Commun (2020) 11:1–11. doi: 10.1038/s41467-020-18513-6 32958743 PMC7505844

[360] Hegazy AN West NR Stubbington MJT Wendt E Suijker KIM Datsi A. Circulating and tissue-resident CD4+ T cells with reactivity to intestinal microbiota are abundant in healthy individuals and function is altered during inflammation. Gastroenterology (2017) 153:1320–1337.e16. doi: 10.1053/J.GASTRO.2017.07.047 28782508 PMC5687320

[361] Vento-Tormo R Efremova M Botting RA Turco MY Vento-Tormo M Meyer KB. Single-cell reconstruction of the early maternal–fetal interface in humans. Nature (2018) 563:347–53. doi: 10.1038/s41586-018-0698-6 PMC7612850 30429548

[362] Chen Y-L Gomes T Hardman CS Vieira Braga FA Gutowska-Owsiak D Salimi M. Re-evaluation of human BDCA-2+ DC during acute sterile skin inflammation. J Exp Med (2020) 217:e20190811. doi: 10.1084/JEM.20190811 31845972 PMC7062525

[363] McMurray JL von Borstel A Taher TE Syrimi E Taylor GS Sharif M. Transcriptional profiling of human Vδ1 T cells reveals a pathogen-driven adaptive differentiation program. Cell Rep (2022) 39:110858. doi: 10.1016/J.CELREP.2022.110858 35613583 PMC9533230

[364] Domínguez Conde C Xu C Jarvis LB Rainbow DB Wells SB Gomes T. Cross-tissue immune cell analysis reveals tissue-specific features in humans. Science (2022) 376:6594. doi: 10.1126/SCIENCE.ABL5197 PMC7612735 35549406

[365] Reynolds G Vegh P Fletcher J Poyner EFM Stephenson E Goh I. Developmental cell programs are co-opted in inflammatory skin disease. Science (2021) 371:6527. doi: 10.1126/SCIENCE.ABA6500 PMC7611557 33479125

[366] Suo C Dann E Goh I Jardine L Kleshchevnikov V Park JE. Mapping the developing human immune system across organs. Science (2022) 376:6597. doi: 10.1126/SCIENCE.ABO0510 PMC7612819 35549310

